# Transmission of Hepatitis B and D Viruses in an African Rural Community

**DOI:** 10.1128/mSystems.00120-18

**Published:** 2018-09-18

**Authors:** Carlos Augusto Pinho-Nascimento, Martin W. Bratschi, Rene Höfer, Caroline Cordeiro Soares, Louisa Warryn, Jūlija Pečerska, Jacques C. Minyem, Izabel C. N. P. Paixão, Marcia Terezinha Baroni de Moraes, Alphonse Um Boock, Christian Niel, Gerd Pluschke, Katharina Röltgen

**Affiliations:** a Swiss Tropical and Public Health Institute, Molecular Immunology, Basel, Switzerland; b University of Basel, Basel, Switzerland; c Laboratory of Molecular Virology, Biology Institute, Fluminense Federal University, Niterói, Brazil; d Jena-Optronik GmbH, Jena, Germany; e Laboratory of Molecular Virology, Oswaldo Cruz Institute, Fiocruz, Rio de Janeiro, Brazil; f Department of Biosystems Science and Engineering, ETH Zürich, Basel, Switzerland; g Oswaldo Cruz Institute, Fiocruz, Laboratory of Comparative and Environmental Virology, Rio de Janeiro, Brazil; h FAIRMED, Yaoundé, Cameroon; University of California, Irvine

**Keywords:** hepatitis B virus, molecular epidemiology, transmission

## Abstract

According to the World Health Organization (WHO), an estimated 257 million people worldwide are chronically infected with hepatitis B virus (HBV), with approximately 15 million of them being coinfected with hepatitis D virus (HDV). To investigate the prevalence and transmission of HBV and HDV within the general population of a rural village in Cameroon, we analyzed serum samples from most (401/448) of the villagers. HBV surface antigen (HBsAg) was detected in 54 (13.5%) of the 401 samples, with 15% of them also containing anti-HDV antibodies. Although Cameroon has integrated HBV vaccination into their Expanded Program on Immunization for newborns in 2005, an HBsAg carriage rate of 5% was found in children below the age of 5 years. Of the 54 HBsAg-positive samples, 49 HBV pre-S/S sequences (7 genotype A and 42 genotype E sequences) could be amplified by PCR. In spite of the extreme geographical restriction in the recruitment of study participants, a remarkable genetic diversity within HBV genotypes was observed. Phylogenetic analysis of the sequences obtained from PCR products combined with demographic information revealed that the presence of some genetic variants was restricted to members of one household, indicative of intrafamilial transmission, which appears to take place at least in part perinatally from mother to child. Other genetic variants were more widely distributed, reflecting horizontal interhousehold transmission. Data for two households with more than one HBV-HDV-coinfected individual indicate that the two viruses are not necessarily transmitted together, as family members with identical HBV sequences had different HDV statuses.

**IMPORTANCE** This study revealed that the prevalence of HBV and HDV in a rural area of Cameroon is extremely high, underlining the pressing need for the improvement of control strategies. Systematic serological and phylogenetic analyses of HBV sequences turned out to be useful tools to identify networks of virus transmission within and between households. The high HBsAg carriage rate found among children demonstrates that implementation of the HBV birth dose vaccine and improvement of vaccine coverage will be key elements in preventing both HBV and HDV infections. In addition, the high HBsAg carriage rate in adolescents and adults emphasizes the need for identification of chronically infected individuals and linkage to WHO-recommended treatment to prevent progression to liver cirrhosis and hepatocellular carcinoma.

## Abstract (precis)

This study revealed that the prevalence of HBV and HDV in a rural area of Cameroon is extremely high, underlining the pressing need for the improvement of control strategies. Systematic serological and phylogenetic analyses of HBV sequences turned out to be useful tools to identify networks of virus transmission within and between households. The high HBsAg carriage rate found among children demonstrates that implementation of the HBV birth dose vaccine and improvement of vaccine coverage will be key elements in preventing both HBV and HDV infections. In addition, the high HBsAg carriage rate in adolescents and adults emphasizes the need for identification of chronically infected individuals and linkage to WHO-recommended treatment to prevent progression to liver cirrhosis and hepatocellular carcinoma.

## INTRODUCTION

Despite being entirely vaccine preventable, hepatitis B virus (HBV) infection remains a serious public health concern, with estimates of 257 million chronic HBV surface antigen (HBsAg) carriers worldwide (1). Without treatment, disease will progress to liver cirrhosis and hepatocellular carcinoma in 15 to 40% of the chronically infected patients. According to WHO figures, about 60 million HBsAg carriers live in the WHO African Region, the majority of whom are unaware of their infection and, thus, constitute a reservoir for the inadvertent spread of the virus to others (2, 3). Transmission of HBV in areas of high endemicity commonly occurs perinatally from mother to child or horizontally by exposure to infected blood or other body fluids. In African settings, the predominant route of HBV transmission appears to be horizontal, in particular, from child to child during the first 5 years of life due to close interaction with infected household contacts and playmates (1). HIV-HBV coinfection seems to lead to an increase in the risk of mother-to-child transmission (3). Occult HBV infection (OBI), characterized by the presence of very low levels of HBV DNA in blood and liver and undetectable HBsAg levels, poses a significant risk to individuals receiving blood transfusions or tissue transplants (4).

In a recent systematic review and meta-analysis on the prevalence of HBV infection in Cameroon, an overall pooled HBsAg carriage rate of 11% was reported, with a higher prevalence in rural (13%) than in urban (9%) areas (5). Seroprevalence rates in rural areas of Cameroon were reported for pregnant women (6, 7), Pygmy groups (8), and HIV patients (9), whereas information on the burden of HBV in the general population, including age-specific prevalence rates, is limited. The impact of perinatal versus horizontal HBV transmission in African countries where the disease is hyperendemic, like Cameroon, remains controversial (6, 7, 10, 11).

HBV isolates from around the world have been classified into nine genotypes (A to I), most of which show a more-or-less distinct geographical distribution (12). In sub-Saharan Africa, a predominance of genotypes A (HBV/A) and E (HBV/E) has been reported (13–15). While HBV/E is characterized by comparatively low genetic diversity (14), a new classification scheme for HBV/A consisting of subgenotypes A1, A2, and A4 (previously referred to as A6) as well as quasi-subgenotype A3 (qA3; previously subdivided into A3, A4, A5, and A7) was proposed (16, 17).

The HBV genome consists of a circular, partly double-stranded DNA of approximately 3,200 nucleotides and comprises four partially overlapping open reading frames (ORFs) encoding the surface proteins (pre-S/S; corresponding to the HBsAg), the HBeAg, and the core protein (pre-C/C), the polymerase (P), and the regulatory protein (X). The pre-S/S ORF encodes three different, structurally related envelope proteins, which are synthesized from alternative initiation codons and are referred to as large (L), middle (M), and small (S) proteins, respectively (18). The pre-S/S region is the most variable part of the HBV genome (19) and is therefore the most commonly analyzed region for phylogenetic investigations.

According to WHO estimates, approximately 15 million of all HBsAg carriers worldwide are chronically coinfected with hepatitis D virus (HDV) (20), a defective virus that requires HBsAg to establish an infection. HDV infection occurs either simultaneously with HBV or through superinfection of an HBV-positive individual. Whereas clearance of both viruses is a common outcome of simultaneous infection, the majority of patients with superinfection progress to chronic HDV and hepatitis (21). The prevalences of HDV in Africa vary geographically and are particularly high in West and Central African countries (22). In these settings, transmission of HDV through contact with the blood or other body fluids of an infected person is common (23), while the risk of vertical spread seems to be low (20). Recent data on the HDV seroprevalence among HBsAg carriers in Cameroon ranged from 11% (24) to 18% (25). HDV exhibits a high degree of genetic heterogeneity and has been classified into eight different clades (1 to 8) (26), of which clade 1 has a worldwide distribution (27). Only a few HDV genotyping studies have been conducted in Africa. One of them reports a cocirculation of several clades (HDV-1, HDV-5, HDV-6, and HDV-7) in Cameroon (25). HDV has a circular RNA genome of approximately 1,700 nucleotides, containing a single ORF encoding the hepatitis delta antigen (HDAg) (28).

The purpose of the present study was to investigate the prevalence and genetic diversity of HBV and HDV in the general population of a remote rural village in Cameroon. By combining these data with available demographic information on the study population, and by assuming that high similarity between HBV or HDV sequences from different individuals is strongly indicative of a related source of infection, intra- and interfamilial patterns of the transmission of these viruses were analyzed.

## RESULTS

### HBsAg and anti-HDV serum antibody positivity in the study population.

**FIG 1:**
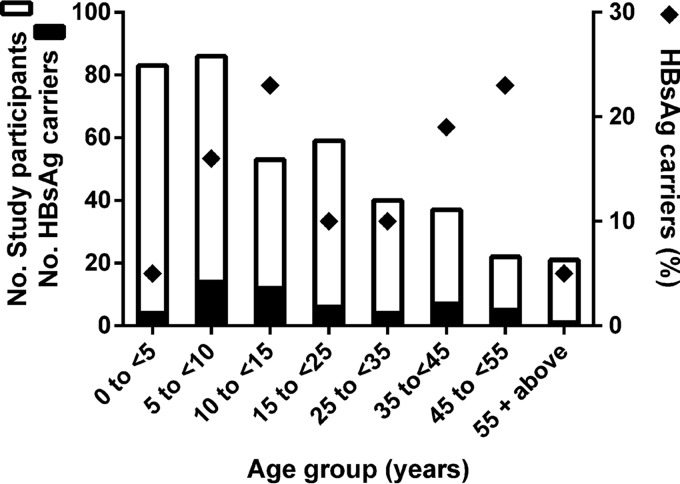
Age distribution of HBsAg carriers among study participants of Mbandji 2. A stacked graph illustrating the number of HBsAg carriers among the total number of study participants for each age group (left *y* axis) is shown. Diamonds represent the percentage of HBsAg carriage for each age group (right *y* axis).

Serum samples from 401 of the 448 inhabitants (living in 88 households) of the village Mbandji 2 in the Bankim Rural Health Area of Cameroon were available for assessing HBV and HDV infection prevalence. Of the 401 individuals, 222 were children under the age of 15 years. An enzyme-linked immunosorbent assay (ELISA) revealed that 13.5% of all sera (54/401) were HBsAg positive. Between different age groups, HBsAg positivity was highly varied, with the lowest prevalence rates recorded for young children (<5 years) and the elderly population (≥55 years) (Fig. 1). There was no marked gender difference in positivity, as 12.6% (26/206) of the male and 14.4% (28/195) of the female participants tested positive. Fifteen percent (8/54) of the sera positive for HBsAg also contained anti-HDV antibodies. Five of the HBV-HDV-coinfected individuals were adults (one male of unknown age, two males aged 28 and 40 years, and two females aged 39 and 50 years), and three were children 9, 11, and 14 years of age.

### Phylogenetic analysis of the circulating HBV and HDV variants.

**FIG 2:**
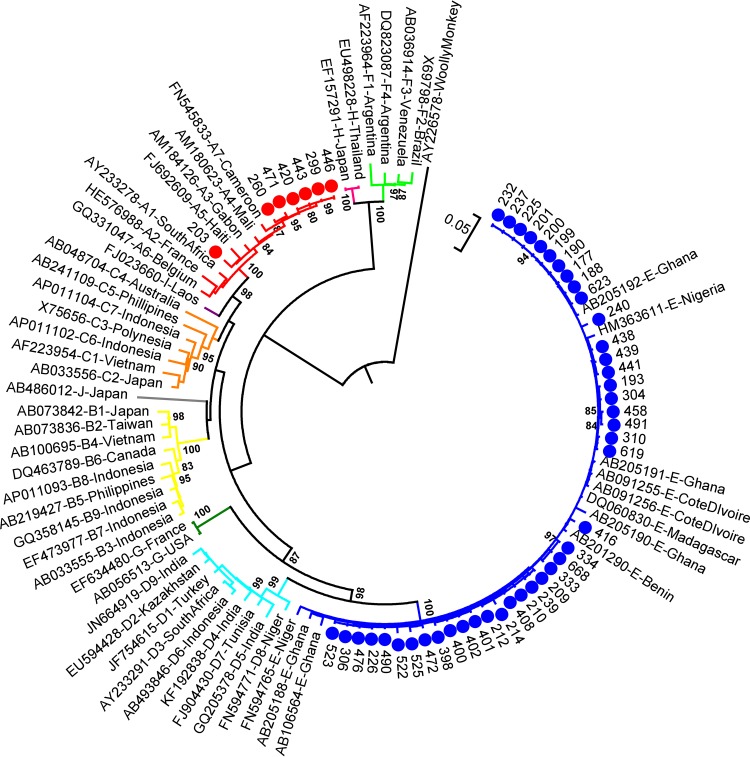
Phylogenetic reconstruction of HBV pre-S/S sequences. A maximum-likelihood phylogenetic tree of the 49 HBV pre-S/S sequences obtained in this study together with 53 publicly available sequences covering all HBV genotypes was constructed under the general time-reversible (GTR) model +G +I embedded in MEGA 6.0. Of the 49 Mbandji 2 sequences, 42 (blue dots) and 7 (red dots) clustered with HBV/E and HBV/A isolates, respectively. The tree was drawn to scale, with branch lengths measured as the number of substitutions per site. The tree was rooted using the sequence of a woolly monkey HBV as an outgroup. There were a total of 1,212 positions in the final data set. Bootstrap values (≥80%) are shown at branch nodes.

HBV pre-S/S region sequences could be amplified from 91% (49/54) of the HBsAg ELISA-positive sera. To examine the position of the genetic variants circulating in Mbandji 2 within the global HBV phylogeny, these sequences were compared with a representative selection of HBV sequences retrieved from GenBank (Fig. 2). While 86% (42/49) of the Mbandji 2 sequences clustered with HBV/E isolates, 14% (7/49) were closely related to HBV/A isolates. The mean genetic distance of the 42 HBV/E pre-S/S sequences was lower (0.7% ± 0.1) than that of the seven HBV/A sequences (1.2% ± 0.2%). HBV whole-genome sequences could be amplified from 72% (39/54) of the HBsAg ELISA-positive samples. Results of phylogenetic analyses of these whole genomes are consistent with results obtained with the pre-S/S sequences (see Fig. S1 in the supplemental material).

10.1128/mSystems.00120-18.1 FIG S1 Phylogenetic reconstruction of HBV whole-genome sequences from Mbandji 2 and of worldwide origin. A maximum-likelihood phylogenetic tree of 35 HBV/E (blue dots) and 4 HBV/A (red dots) sequences from Mbandji 2 together with 53 publicly available sequences of worldwide origin (accession numbers are given in the graph) covering all HBV genotypes was constructed under the GTR model +G +I embedded in MEGA 6.0. The tree is drawn to scale, with branch lengths measured according to the number of substitutions per site. The tree was rooted using the sequence of a woolly monkey HBV as an outgroup. Bootstrap values (≥80%) are shown at branch nodes. Download FIG S1, TIF file, 0.6 MB. Copyright © 2018 Pinho-Nascimento et al. 2018 Pinho-Nascimento et al. This content is distributed under the terms of the Creative Commons Attribution 4.0 International license.

**FIG 3:**
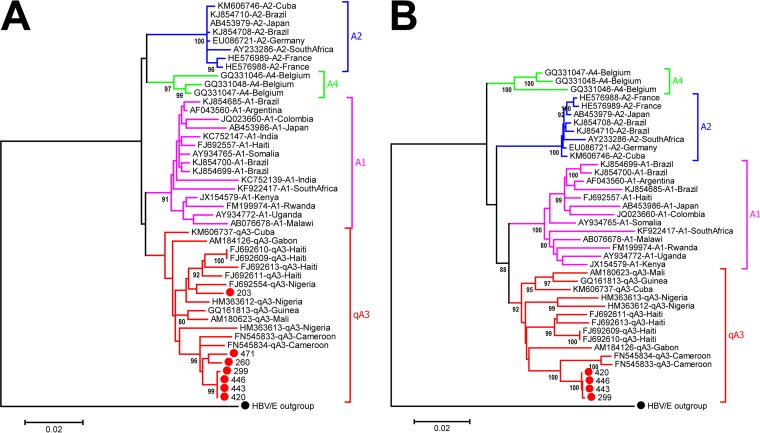
High-resolution phylogeny of HBV/A sequences. Maximum-likelihood phylogenetic trees of HBV/A pre-S/S (A) and whole-genome (B) sequences from Mbandji 2 (red dots) together with publicly available sequences covering the described HBV/A subgenotypes were constructed in MEGA 6.0. (A) All of the seven HBV/A pre-S/S sequences of this study clustered with strains of quasi-subgenotype A3 (qA3). Distances were calculated using the Kimura 2-parameter model +G. There were a total of 1,206 positions in the final data set. (B) Classification of the Mbandji 2 HBV/A sequences into qA3 was reconfirmed by whole-genome analysis. Distances were calculated using the Kimura 2-parameter model +G +I. There were a total of 3,221 positions in the final data set. Both trees were drawn to scale, with branch lengths measured as the number of substitutions per site, and were rooted using the sequence of an HBV/E strain as an outgroup (black dot). Bootstrap values (≥80%) are shown at branch nodes.

High-resolution phylogenetic analysis of the Mbandji 2 HBV/A sequences demonstrated that all of the seven pre-S/S sequences of this study grouped with strains belonging to quasi-subgenotype A3 (Fig. 3A). Classification into quasi-subgenotype A3 was confirmed by whole-genome analysis of the four complete HBV/A genome sequences obtained (Fig. 3B).

**FIG 4:**
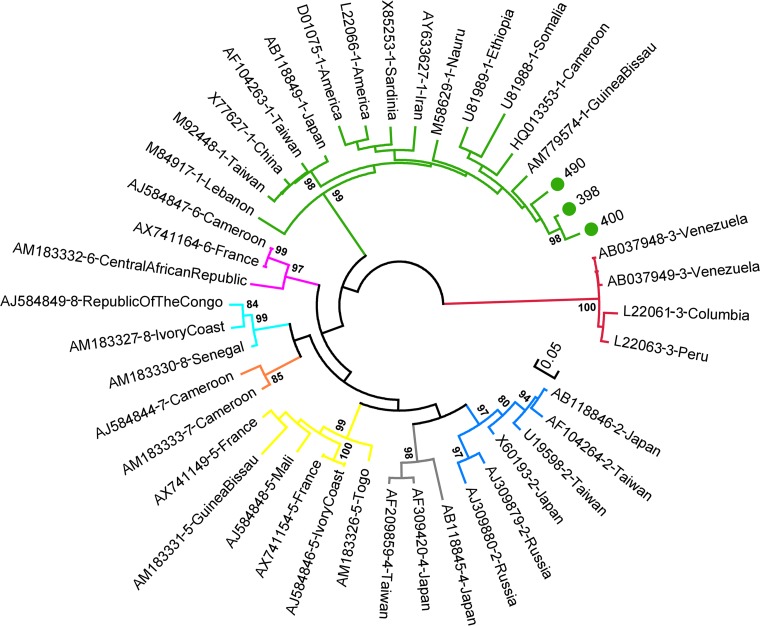
Phylogenetic reconstruction of HDV sequences. An unrooted maximum-likelihood phylogenetic tree of the 3 HDV sequences obtained in this study (green dots) and 41 publicly available sequences covering all currently described HDV genotypes (1 to 8) was constructed with the GTR model +G embedded in MEGA 6.0. The three Mbandji 2 sequences clustered with strains of HDV genotype 1. The tree was drawn to scale, with branch lengths measured as the number of substitutions per site. There were a total of 365 positions in the final data set. Bootstrap values (≥80%) are shown at branch nodes.

For HDV, amplification of a 360-bp fragment of the small hepatitis D (sHD) gene region succeeded for three of the eight samples in which anti-HDAg antibodies were detected. Phylogenetic reconstruction together with publicly available sequences of different HDV clades showed a clustering of the three sequences with HDV clade 1 isolates (Fig. 4).

### Phylogeography and evidence for familial transmission of HBV and HDV.

**FIG 5:**
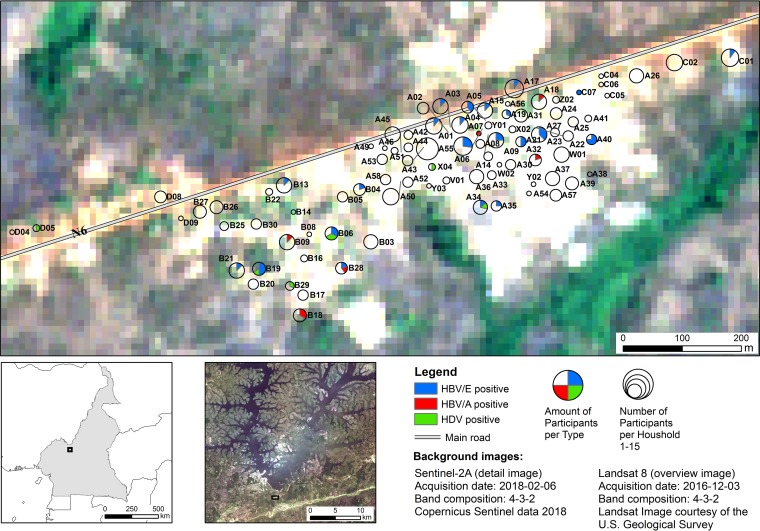
Geographical distribution of HBV/A, HBV/E, and HDV infections in Mbandji 2. Locations of the 88 households participating in the study are displayed in the form of pie charts, with the pies corresponding in size to the numbers of study participants. Individuals identified as being infected with HBV/A (red) and HBV/E (blue) or coinfected with HBV/E and HDV (green) are represented as slices in the pie charts according to their numerical proportion. No household location is shown for study participant 490, coinfected with HBV/E and HDV, because no demographic information was available. Overview images of Cameroon (lower left) and the Mapé Basin (lower middle) are shown to illustrate the geographical location of the study area (black rectangle). The background images, courtesy of ESA Sentinel and the U.S. Geological Survey, are in the public domain.

**FIG 6:**
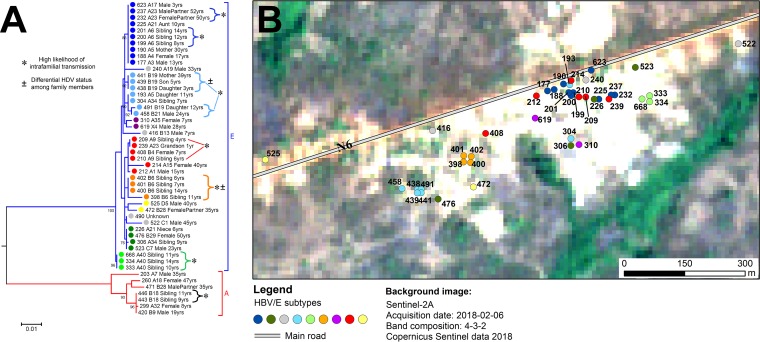
Phylogeographic and transmission analysis of HBV in the population of Mbandji 2. (A) A maximum-likelihood phylogenetic tree of the 42 HBV/E and 7 HBV/A pre-S/S sequences was constructed using the Kimura 2-parameter model +G to illustrate inter- and intrafamilial HBV transmission in the village. Individuals are marked with dots colored according to different branches of the phylogenetic tree, and additional demographic information, such as participant ID, household ID, gender, family relationships (for households with sequence information from several HBsAg carriers), and age, is shown. The tree was drawn to scale, with branch lengths measured as the number of substitutions per site. There were a total of 1,209 positions in the final data set. Bootstrap values (≥80%) are shown at branch nodes. Asterisks indicate households in which members with identical or highly similar HBV sequences were identified (high likelihood of intrafamilial transmission). Plus or minus signs highlight households in which family members with identical HBV sequences had differential HDV statuses. (B) The residence of each of the study participants (except for participant 490, for whom no demographic information was available) with HBV/E sequence information is displayed according to the color of the respective dots in the branches of the phylogenetic tree. The background image, courtesy of ESA Sentinel, is in the public domain.

The geographical distribution of the 88 studied households is depicted in a map of Mbandji 2 in the form of pie charts, corresponding in size to the numbers of study participants (Fig. 5). The 42 study participants infected with variants of HBV/E resided in 25 households, 9 of which were inhabited by more than one HBV/E infected individual. In order to study the spatial pattern of HBV/E variants within the village, a phylogeny of the 49 pre-S/S sequences was constructed (Fig. 6A) (HBV/A sequences were used as an outgroup). Dots representing the residential homes of each individual with HBV/E sequence information in a map of Mbandji 2 (Fig. 6B) are colored according to the corresponding branches in the HBV/E phylogenetic tree in Fig. 6A, in which information on participant identifiers (IDs), household IDs, gender, family relationships, and age is also provided. The spatial analysis revealed that in most cases, the HBV/E sequences from infected individuals living in the same households clustered together (Fig. 6), indicating prevailing intrahousehold transmission of HBV.

HBV/E pre-S/S sequences with high similarity were found in six of the nine households inhabited by more than one HBV/E-infected individual. This included two genetic clusters with completely identical sequences each: one (cluster E1 in Table 1; light green in Fig. 6) consisting of sequences from three siblings living in household A40 and the other (cluster E2 in Table 1; dark blue in Fig. 6) comprising sequences from three siblings living in household A6 and a couple residing in household A23, as well as five additional individuals from five other households. Another genetic cluster (cluster E3 in Table 1; light blue in Fig. 6) consisted of sequences from a mother with her three children resident in household B19 as well as sequences from three additional individuals from three other households. While the sequences of the mother and her two youngest children were identical and contained two unique single-nucleotide polymorphisms (SNPs) that were not present in any other sequence in the entire data set, the sequence of the oldest daughter exhibited six additional SNP differences, two of which were uniquely found in her sequence, whereas four also occurred in the almost identical sequence of her cousin living in household B21. It is noteworthy that the mother tested HDV positive but that her three children were HDV negative. Sequences from two siblings living in household A9 (cluster E4 in Table 1; red in Fig. 6) differed by only a single SNP. They clustered together with the sequences of four additional individuals from other households. The mother of the two siblings was HBsAg negative. Sequences of three siblings (cluster E5 in Table 1; orange in Fig. 6) living in household B6 were completely identical. The sequence of a fourth sibling living in this household exhibited three unique SNPs and a 15-bp deletion that was found only in this isolate. The sequences of all four siblings shared two unique SNPs that were not present in any other sequence of the whole data set. Particularly notable is that the two female siblings were identified to be coinfected with HDV but that the two male siblings tested anti-HDV antibody negative.

**TABLE 1:**
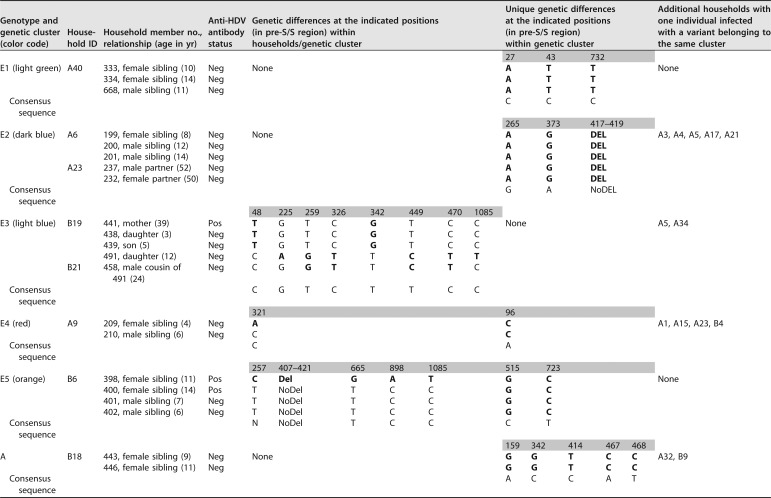
Transmission of HBV and HDV within households^*a*^

a HBV/E and HBV/A pre-S/S sequences with high similarity (genetic clusters) were found in members of the same households. To illustrate the composition of the genetic clusters listed in the first column of the table (the color code corresponds to the color scheme in Fig. 6), IDs of affected households, IDs of household members, and their relationships are specified. The presence (or absence) of anti-HDV antibodies in the sera of individuals is indicated. Pos, positive; Neg, negative; Del, deletion; NoDel, no deletion. Genetic differences detected between HBV pre-S/S sequences of household members as well as unique sequence characteristics that were found only in sequences belonging to the same genetic cluster are also listed. To complete the picture, households with only one individual infected with a variant belonging to the same genetic cluster are given in the last column. Boldface indicates genetic differences with respect to the consensus (the nucleotides present in the majority of sequences of this study).

In three of the nine households with more than one HBV/E-infected individual, no particularly close phylogenetic relation was found between sequences of the inhabitants (Fig. 6). Among these households, sequences were derived from (i) a mother (dark blue) living in household A5 with her 11-year-old daughter (light blue), whose sequence was closely related to another child, (ii) an aunt (dark blue) and her niece (dark green) resident in household A21, and (iii) a pair of siblings living in household A34 (light blue and dark green). While one of the siblings was coinfected with HDV, the other tested anti-HDV antibody negative.

Residences of the seven individuals from whom HBV/A sequences were obtained are distributed over the entire study area, with two of them living in the same household, B18 (Fig. 5). These two individuals were siblings (9 and 11 years of age) and presented with completely identical sequences (cluster A in Table 1).

In one household (B28), an HBV/E sequence was amplified from the serum of a female and an HBV/A sequence from her male partner.

The eight individuals with HDV infection were all coinfected with HBV/E; their homes (six different households) were located mostly in the southwestern area of the village (Fig. 5). For one of the eight HBV-HDV-coinfected individuals, no demographic information was available.

### Deduced amino acid sequence analysis.

SNP sites among the 42 available HBV/E pre-S/S sequences and the pre-C/C, X, and P sequences of the 35 complete HBV/E genomes are listed in Table S2. Predicted amino acid sequence polymorphisms and deletions are illustrated in Table S3.

Twenty-three of the 35 HBV/E sequences for which full genome data were available were 3,212 nucleotides long. In-frame deletions of 1 to 5 amino acids affecting both the pre-S2 and P regions were detected in the remaining 12 isolates. Deduced amino acid sequences for the pre-S1 region of all 42 HBV/E pre-S/S sequences showed the characteristic features described for genotype E isolates (29), including a length of 118 amino acids due to a single amino acid deletion in the amino terminus, the signature motif Leu^3^SerTrpThrValProLeuGluTrp^11^, and a methionine at position 83, which introduces a new translational start codon. Interestingly, another methionine at pre-S1 position 86 was present in 17 of the 42 sequences. Substitutions leading to the loss of the pre-S2 start codon, with consequent abolishment of the M protein synthesis, were detected in the sequences of two individuals (study participants 214 and 522). Ten isolates, which constitute the dark-blue branch of the phylogenetic tree (Fig. 6A), had a single amino acid deletion at pre-S2 position 22. Two isolates from individuals living in two different households contained unique amino acid deletions of 4 and 5 amino acids at pre-S2 positions 19 to 22 (study participant 240) and 18 to 22 (study participant 398), respectively. In the S region, all but one of the sequences had a Thr^57^ residue, like the majority of isolates from northwestern Africa. In contrast, an Ile^57^ residue present in the majority of HBV/E isolates from southwestern Africa (29) was detected in one sequence belonging to a 7-year-old child (study participant 416), who was the only HBsAg-positive individual in his household. Amino acid substitutions in the “a” determinant of the major hydrophilic region, associated with immune escape, consisting of amino acids 124 to 147 deduced from the S gene sequence (30) were identified in three sequences at positions L127I (study participant 398), L127P (study participant 416), and S140L (study participant 240). Analysis of the predicted amino acid sequences of the 35 HBV/E whole-genome sequences revealed that the amino acid sequence deduced from the pre-C/C region was particularly conserved, except for the sequences of study participants 240, 472, and 522, which contained a number of amino acid replacements in this region (Table S3). A stop codon mutation affecting amino acid sequence position W28* deduced from the pre-C gene region (nucleotide position G1896A in the genome sequence), abolishing HBeAg production, was detected in two sequences (study participants 472 and 522).

The four HBV/A sequences with complete genome information were 3,221 nucleotides long with a 2-amino-acid insertion at the carboxy terminus of the core protein, characteristic of HBV/A isolates. Of the only three SNPs detected among the four isolates, one was nonsynonymous (amino acid position 244 in the P region), whereas the other two were synonymous. One of the two synonymous SNPs found in three of the four sequences was the silent G1888A (nucleotide position in the genome sequence) mutation, which has previously been described as a unique characteristic of subgenotype A1 (31).

SNPs among the seven HBV/A pre-S/S sequences as well as the corresponding deduced amino acids are listed in Table S2. All predicted polymorphic amino acid positions detected in the pre-S/S region are illustrated in Table S3. The deduced amino acid sequence from the pre-S1 gene region of six sequences was found to be 119 amino acids long, while that of the seventh isolate, belonging to a 35-year-old male (study participant 203) who was the only participant of his household, contained 120 amino acids. His sequence also contained a mutation leading to the loss of the pre-S2 start codon. No deletions as well as no mutations in the “a” determinant of the major hydrophilic region were detected in any of the HBV/A pre-S/S sequences.

No mutations in the polymerase region known to be relevant for phenotypic resistance to the five antiretroviral drugs lamivudine, adefovir, entecavir, tenofovir, and telbivudine were found in any of the HBV sequences.

Based on the S region amino acid sequence algorithms, Arg^122^, Lys^160^, and Leu/Ile^127^, which were found in 41 HBV/E sequences, and Arg^122^, Lys^160^, Pro^127^, Gly^159^, and Ser^140^, which were found in 1 HBV/E sequence (study participant 416), all HBV/E isolates of this study were classified as serotype *ayw4*. All HBV/A isolates were predicted to belong to serotype *ayw1* based on their Arg^122^, Lys^160^, Pro^127^, and Ala^159^ amino acid pattern (32).

### Detection of OBI and placement of sequences in the HBV phylogeny.

In order to detect OBI in the study population, nucleic acids were extracted from the sera of 149 household contacts of the 54 HBsAg-positive individuals. Pre-S/S amplification products were obtained from 19 of the 149 sera. Nucleotide sequencing and phylogenetic analysis revealed that the 19 OBI sequences were highly similar to the sequences of the 49 HBsAg-positive individuals, with 17/19 clustering with HBV/E sequences and the remaining 2 being closely related to the HBV/A sequences identified in this study (Fig. S2). Of the 17 occult HBV/E sequences, 5 were obtained from members of household A6. One of the five sequences, derived from the mother, was identical to the sequences of her three HBsAg-positive children, whereas four clustered with other sequences. Of the remaining 12 occult HBV/E sequences, 5 were highly similar to the sequences of their household contacts living in households A3, A9, A17, A19, and A23, 3 (from household A15) were located on different branches of the tree, and 4 (from households A4, A18, A35, and B9) showed no particularly close phylogenetic relation to the sequences of their household contacts. HBsAg-positive household contacts living in A18 and B9 were infected with HBV/A.

10.1128/mSystems.00120-18.2 FIG S2 Phylogenetic reconstruction of Mbandji 2 HBV sequences from HBsAg-positive individuals and individuals with OBI. A maximum-likelihood phylogenetic tree of the 42 HBV/E and 7 HBV/A (whole-genome and pre-S/S) sequences from HBsAg-positive individuals as well as 17 HBV/E and 2 HBV/A (pre-S/S) sequences from individuals with OBI was constructed using the GTR model +G to illustrate the phylogenetic positions of the occult HBV/E and HBV/A sequences. To analyze whole-genome and pre-S/S sequences at the same time, pre-S/S sequences were complemented with unknown nucleotides (N) for regions of the complete genome flanking the pre-S/S sequences. Occult HBV sequences are in red. Individuals are marked with multicolored dots according to different branches of the phylogenetic tree, and additional demographic information, such as participant ID, household ID, gender, family relationships, and age, are shown. The tree was drawn to scale, with branch lengths measured according to the number of substitutions per site. Bootstrap values (≥80%) are shown at branch nodes. Download FIG S2, TIF file, 2 MB. Copyright © 2018 Pinho-Nascimento et al. 2018 Pinho-Nascimento et al. This content is distributed under the terms of the Creative Commons Attribution 4.0 International license.

Of the two occult HBV/A sequences, only one was highly similar to the sequence of the HBsAg-positive household contact living in A32, whereas the other HBsAg-positive household contact residing in B4 was infected with HBV/E.

Highly similar phylogenetic relations were obtained by means of Bayesian analysis (Fig. S3).

10.1128/mSystems.00120-18.3 FIG S3 Rooted maximum-clade-credibility tree of Mbandji 2 HBV sequences from HBsAg-positive individuals and individuals with OBI summarized from the Bayesian analysis posterior tree sample. To analyze whole-genome and pre-S/S sequences at the same time, pre-S/S sequences were complemented with unknown nucleotides (N) for regions of the complete genome flanking the pre-S/S sequences. Tree branch length is measured in years. The gray bars indicate the 95% highest posterior density interval of the particular node age (bottom axis). (A) Linear scale; (B) log scale with internal node labels showing the posterior probability of the underlying clade in the tree sample. Download FIG S3, PDF file, 0.4 MB. Copyright © 2018 Pinho-Nascimento et al. 2018 Pinho-Nascimento et al. This content is distributed under the terms of the Creative Commons Attribution 4.0 International license.

In contrast to the S regions of HBsAg-positive individuals, all of which had a Val^106^ residue, both of the OBI HBV/A sequences had Ile^106^. No amino acid variation characteristic for all OBI HBV/E sequences was detected.

## DISCUSSION

Despite the high endemicity of chronic HBV infection and HBV-HDV coinfection in West and Central Africa, data on the epidemiology of these viruses in the region are scarce, and those available are mostly from urban areas. Here, we investigated the prevalence and genetic diversity of HBV and HDV in a remote rural population of the Mbandji 2 community of Cameroon, for which detailed demographic information was available to study familial relationships of transmission. A high prevalence of HBsAg carriers (13.5%) and HBV-HDV coinfections (15%) was detected among inhabitants of the Mbandji 2 village, mainly in individuals born in the era before Cameroon integrated the HBV vaccine into its Expanded Program on Immunization for newborns in 2005 (33). However, the HBsAg prevalence rate in children below the age of 5 years was still high (5%), which might be explained by poor access to medical care. In rural African settings, prevention of mother-to-child transmission, requiring a combination of routine antenatal screening, antiviral drug treatment during pregnancy, and the integration of HBV birth dose vaccination, needs to be implemented and improved (3). A lower HBV prevalence in the elderly population (>55 years of age) than in younger adults, as observed in our study population, has also been described in another study (34). This may be associated with the high mortality rate of elderly individuals infected with HBV, presumably due to immunosenescence combined with a high prevalence of other chronic conditions accumulated during their lifetimes (35, 36). The extremely high HBsAg carrier rate of >20% in some age groups demonstrates that public health efforts are required to diagnose infections in the community and to ensure access to treatment. None of the known drug resistance mutations was detected in any of the analyzed HBV sequences, which might be explained by the lack of selection pressure due to the limited use of antiviral drugs in this region.

By analyzing genomic sequences, evidence of familial transmission of HBV became apparent for 7 of the 11 households in which more than one HBV-positive individual was identified; identical or almost identical HBV sequences were isolated from infected household members. It has been reported previously that apart from perinatal transmission, horizontal transmission during early childhood may be a common mechanism of HBV infection in sub-Saharan Africa (7, 10, 37). In our study, six households in which siblings were infected with highly similar HBV/E or HBV/A sequences were identified. In one of these (household B19), perinatal transmission from a mother to her three children is most likely. That it is, in any case, an example of intrafamilial transmission is proven by a genomic fingerprint; the viral sequences of the mother and her two youngest children contained two unique SNPs that were not found in any other sequence. With respect to the involvement of the mother in intrafamilial transmission, the situation is less clear for the other five households, in which the mother either had OBI (household A6), was HBsAg negative (household A9), or did not participate in the study (households A40, B6, and B18). Interestingly, the HBV pre-S/S sequence derived from the mother with OBI of household A6 was identical to the sequences of three of her children. Unique SNPs in almost-identical pre-S/S sequences of family members were also identified in households A40 and B6.

Routes of HDV and HBV transmission were reported to be similar. Apart from sexual contact, inadvertent intrafamilial spread of HDV seems to be common in regions of high prevalence. The risk of perinatal transmission of HDV is considered to be low (38). Anti-HDV IgG levels, which indicate exposure to HDV and persist long term, even after viral clearance, were detected in eight study participants. In all three cases in which HDV infections occurred in households with more than one HBsAg carrier, HDV coinfection affected only part of the HBV-positive inhabitants. Interestingly, in two of these (households B6 and B19), individuals with completely identical HBV sequences had different HDV statuses. While in one family (household B6), HBV-HDV coinfection was identified in two of four siblings, in another family (household B19), the mother, but none of her three HBV-infected children, was coinfected. Since the sensitivity and specificity of the applied anti-HDAg antibody kit was reported to be very high (39), these results suggest that HDV is not necessarily transmitted together with HBV from a coinfected donor. However, the possibility that the two siblings (household B6) and the mother (household B19) acquired their HDV infections from different sources cannot be ruled out completely.

Of the 49 individuals from whom HBV pre-S/S sequences were obtained in this study, 7 and 42 belonged to genotypes A and E, respectively. The three analyzed HDV sequences showed high similarity to other publicly available HDV clade 1 sequences. Cocirculation of HBV/A and HBV/E genotypes (15) as well as a predominance of HDV clade 1 infections (25) in Cameroon have been reported previously. Intragenotype mean genetic distances of the analyzed HBV/A and HBV/E pre-S/S sequences were determined to be 1.2% and 0.7%, respectively. Within the geographically very confined study population, the mutation rate appeared to be relatively low, since completely identical genomic sequences were found in inhabitants of the same households and even in individuals living in different households (dark-blue branch of the phylogenetic tree). A maximum of six unique SNPs were observed between the sequences of family members with apparently linked transmission (household B19). In this household, a mother most likely transmitted her HBV variant perinatally to her three children, which were at the time of blood collection 3, 5, and 12 years of age. While the mother and her two younger children exhibited completely identical sequences, the HBV strain of her 12-year-old daughter had accumulated six unique SNPs.

In two of the HBV/E sequences and in one HBV/A sequence, we detected mutations leading to the abolishment of the pre-S2 start codon. In addition, deletions within the pre-S2 region of 1 to 5 amino acids were identified in 19 HBV/E sequences (12 from HBsAg-positive individuals and 7 from individuals with OBI). The pre-S2 gene is known as the most varied region of the HBV genome, and the emergence of pre-S2 mutants is a frequent event that may occur spontaneously or as a consequence of immune or drug pressure (19). Several lines of evidence indicate that deletions in this region tend to accumulate during later stages of persistent HBV infection and are associated with different severe forms of acute and chronic liver disease (19). In our study, the occurrence of a single amino acid deletion in completely identical sequences from 16 participants may reflect frequent transmission of this variant. The site of this deletion as well as the one in three sequences with unique 4- or 5-amino-acid deletions involves a region with known B cell epitopes (40) and is therefore indicative of the emergence of HBV immune escape variants. The potential correlation between the pre-C mutation G1896A, detected in two sequences of this study, and severity of liver disease remains controversial (41).

The prevalence of HBV, particularly in rural areas of Africa remains very high. Achieving the WHO goals of eliminating HBV (and HDV) as a public health threat by 2030 (2) will require more-effective prevention of new infections via implementation of the HBV birth dose vaccine and improvement of vaccine coverage. Furthermore, treatment protocols for chronic HBV infection suitable for low-income countries (42) will have to be implemented in sub-Saharan Africa.

## MATERIALS AND METHODS

### Study design.

Within the framework of a cross-sectional house-by-house survey for the neglected tropical skin disease Buruli ulcer in the Mapé River Basin (Bankim District) of Cameroon, located in the northwest Adamawa region (43, 44), serum samples from 401 of 448 inhabitants living in 88 different households of the village Mbandji 2 were collected in January 2011. Here, we tested the 401 sera retrospectively for the presence of HBV and HDV infection markers. Nucleic acid extraction and genome sequencing were performed to obtain an overview of the genetic population structure of the viruses in this region. Demographic information on the study population, including age, gender, and GPS coordinates of the participants' residences, recorded during the survey, was extracted to enable a microepidemiological investigation of HBV and HDV infections.

### Ethics statement.

Ethical clearance for the collection and testing of human blood samples was obtained from the Cameroon National Ethics Committee (reference no. Nu172/CNE/SE/201) as well as the Ethics Committee of Basel (EKBB, reference no. 53/11). Written informed consent was obtained from all individuals involved in the study. Parents or guardians provided written consent on behalf of children.

### ELISA testing for the presence of HBV and HDV infection markers.

All serum samples were screened for the presence of HBsAg as an indicator for an HBV infection by using the bioelisa HBsAg3.0 kit (Biokit, Barcelona, Spain) according to the manufacturer’s instructions. Serum samples with a positive HBsAg ELISA result were subsequently analyzed with the ETI-AB-DELTAK-2 ELISA kit (DiaSorin, Saluggia, Italy) by following the manufacturer’s protocol to test for the presence of anti-HDAg antibodies as a marker for HDV infection. Nucleic acids were extracted from all serum samples of HBsAg-positive individuals as well as from sera of household contacts of these individuals to enable the detection of OBI.

### Nucleic acid extraction, PCR, and genome sequencing.

Isolation of viral nucleic acids from 200 µl of each of the serum samples was performed by using the High Pure viral nucleic acid kit (Roche Diagnostics, Mannheim, Germany) in accordance with the manufacturer’s instructions. Total nucleic acids were resuspended in 50 µl Tris-EDTA (TE) buffer. The amplification of HBV whole-genome sequences was attempted with a protocol modified from reference 45 using 4 µl of the nucleic acid template, primers P1 (5′-CCGGAAAGCTTGAGCTCTTCTTTTTCACCTCTGCCTAATCA-3′) and P2 (5′-CCGGAAAGCTTGAGCTCTTCAAAAAGTTGCATGGTGCTGG-3′), and the following PCR profile: denaturation at 94°C for 4 min followed by 11 cycles at 94°C for 40 s, 55°C for 1 min, and 72°C for 3 min; 11 cycles of 94°C for 40 s, 60°C for 1 min, and 72°C for 5 min; 11 cycles of 94°C for 40 s, 62°C for 1 min, and 72°C for 7 min; 11 cycles of 94°C for 40 s, 62°C for 1 min, and 72°C for 9 min; and a final extension step at 72°C for 10 min. Samples for which no DNA could be amplified in the HBV whole-genome PCR were subjected to seminested PCR assays targeting the HBV pre-S/S region (34, 46). Amplification was attempted with 3 µl of the nucleic acid template and primer pairs PS1 (5′-CCATATTCTTGGGAACAAGA-3′) and P3 (5′-AAAGCCCAAAAGACCCACAA-3′) in the first assay round and PS1 and S2 (5′-GGGTTTAAATGTATACCCAAAA-3′) in the second assay round. We also amplified a smaller fragment spanning the S gene using primers PS1a (5′-GGAAAACATCACATCAGGAT-3′) and P3 for the first round of PCR and primers PS1b (5′-AAAATTCGCAGTCCCCAACC-3′) and P3 for the second round. Thermal conditions for all of the described PCR assays included an initial denaturation step at 94°C for 5 min followed by 32 cycles at 94°C for 30 s, 57°C for 30 s, and 72°C for 1 min and a final extension step at 72°C for 10 min. All PCRs were performed using Platinum *Taq* DNA polymerase and supplied reagents (Invitrogen, Carlsbad, CA) in accordance with product recommendations.

For reverse transcription of HDV RNA, 15 µl of the nucleic acid template was heated to 95°C for 5 min before being incubated with deoxynucleoside triphosphates (dNTPs) and primer 1302 (5′-GGATTCACCGACAAGGAGAG-3′) at 70°C for 10 min. In a next step, cDNA synthesis was attempted by adding Moloney murine leukemia virus (M-MLV) reverse transcriptase and M-MLV buffer (Sigma) to the mix and incubating it at 37°C for 50 min, followed by subjection to 94°C for 10 min. For cDNA amplification of a 360-bp fragment of the HDV small HD (sHD) gene sequence, nested PCR was performed with 10 µl of cDNA and primers 1302 and 853 (5′-CGGATGCCCAGGTCGGACC-3′) in the first assay round and 5 µl of the amplicon and primers 5414 (5′-GAGATGCCATGCCGACCCGAAGAG-3′) and 5415 (5′-GAAGGAAGGCCCTCGAGAACAAGA-3′) in the second assay round. Thermal conditions for both of the reactions included an initial denaturation step at 94°C for 2 min followed by 35 cycles of 94°C for 30 s, 55°C for 50 s, and 72°C for 1 min and a final extension step at 72°C for 5 min (47).

All reactions were performed in a TProfessional basic thermocycler (Biometra). PCR products were resolved on 1% agarose gels and were purified with NucleoSpin gel and PCR cleanup kits (Macherey-Nagel, Düren, Germany). Sequencing was done at Macrogen Inc., Europe (Amsterdam, the Netherlands) using primers listed in Table S1.

10.1128/mSystems.00120-18.4 TABLE S1 Primers used for HBV (whole-genome and pre-S/S) and HDV (sHD gene fragment) nucleotide sequencing. All primer sequences were published previously, and references are listed together with primer IDs. Download Table S1, PDF file, 0.1 MB. Copyright © 2018 Pinho-Nascimento et al. 2018 Pinho-Nascimento et al. This content is distributed under the terms of the Creative Commons Attribution 4.0 International license.

10.1128/mSystems.00120-18.5 TABLE S2 SNPs and variations in the predicted HBV/E and HBV/A amino acid sequences obtained in this study. The table is divided into sections a to e, containing sequence information for the pre-S/S region of the 42 HBV/E sequences (a), the pre-C/C, X, and P regions of the 35 HBV/E whole-genome sequences (b to d), and the pre-S/S region of the 7 HBV/A sequences (e). Columns from left to right contain nucleotide and amino acid positions of variable sites, variable nucleotides (as nucleotide 1/nucleotide 2), deduced amino acids at these sites (as amino acid 1/amino acid 2), the number of sequences containing variable positions (nucleotides and amino acids) (as position 1/position 2), and information on amino acid variations due to synonymous (No) or nonsynonymous (Yes) SNPs, as well as the name of the HBV genome region. Different base replacements that occurred at the same site are listed in separate rows, with cells containing redundant information merged. Sites where the replacement of amino acids is dependent on several SNPs in the same codon of a sequence are marked with asterisks. Download Table S2, PDF file, 0.1 MB. Copyright © 2018 Pinho-Nascimento et al. 2018 Pinho-Nascimento et al. This content is distributed under the terms of the Creative Commons Attribution 4.0 International license.

10.1128/mSystems.00120-18.6 TABLE S3 Replacements in predicted HBV/E and HBV/A amino acid sequences obtained in this study. The table is divided into sections a to e, showing amino acid replacements present in the pre-S/S region of the 42 HBV/E and 17 OBI HBV/E sequences (a), the pre-C/C, X, and P regions of the 35 HBV/E whole-genome sequences (b to d), and the pre-S/S region of the 7 HBV/A and 2 OBI HBV/A sequences (e). The consensus shows amino acids present in the majority of sequences of this study. Download Table S3, PDF file, 0.3 MB. Copyright © 2018 Pinho-Nascimento et al. 2018 Pinho-Nascimento et al. This content is distributed under the terms of the Creative Commons Attribution 4.0 International license.

### Phylogenetic analysis of HBV and HDV isolates and inference of HBV serotypes.

Genotyping of HBV was done by analyzing either whole-genome sequences or the pre-S/S gene region. Genotyping of HDV was based on a partial sHD gene region. Sequences were assembled using CodonCode Aligner software version 6.0.2 (CodonCode Corporation) and were aligned with MUSCLE implemented in MEGA version 6.0 (48). Maximum-likelihood phylogenetic analysis was performed with MEGA 6.0 after we inferred the best DNA substitution model for the different data sets. Node support for all constructed phylogenies was assessed with 1,000 bootstrap replicates. Reference sequences included in the phylogenies were retrieved from GenBank, and accession numbers are given in the respective figure legends. MEGA 6.0 was also used to estimate the mean genetic distances among HBV sequences of the same genotype using the Kimura 2-parameter model. Bayesian phylogenetic analysis was performed using the birth-death migration model (BDMM; v0.2.0) (49) within the BEAST2 framework (v2.4.7). For that purpose, we analyzed whole-genome sequences as well as pre-S/S sequences complemented with unknown nucleotides where genetic data were missing. We labeled the samples according to their occult/active status, where the occult cases would not be allowed to transmit. Active cases were allowed to produce both active and occult secondary cases. We applied the general time-reversible (GTR) model with empirical base frequencies as the substitution model and fixed the mean substitution rate to 1.9E^–4^ (50), allowing for some variation in the rate. The Markov chain Monte-Carlo (MCMC) algorithm was run until 60,000,000 states were sampled and 10% of the samples were discarded as burn-in. The trees were logged on every 10,000th MCMC step, and the tree sample was summarized using TreeAnnotator as a maximum-clade-credibility tree, with 20% of samples discarded as burn-in and median heights used as the node heights in the tree.

Previously published mutations in HBV strains associated with “escape,” diminished antibody binding, or phenotypic resistance to antiretroviral drugs were predicted and analyzed using the Geno2pheno[HBV] online tool at http://hbv.geno2pheno.org/index.php.

HBV serotypes were determined on the basis of predicted amino acids at positions 122, 160, 127, 159, and 140 of the HBsAg sequence as described previously (32) using the Web-based HBV Serotyper tool (51), which can be accessed at http://hvdr.bioinf.wits.ac.za/SmallGenomeTools.

### Data availability.

All HBV pre-S/S and whole-genome sequences as well as the sequences of fragments of the sHD gene obtained in this study were deposited in GenBank under accession numbers MG821087 to MG821154, MH580614 to MH580652, and MH595493 to MH595495, respectively.
