# Supplementary figures and images for: Transmission of Hepatitis B and D Viruses in an African Rural Community
Source: mSystems. 2018 Sep 18;3(5):e00120-18. doi: 10.1128/mSystems.00120-18 (PMC6143728; doi:10.1128/mSystems.00120-18)

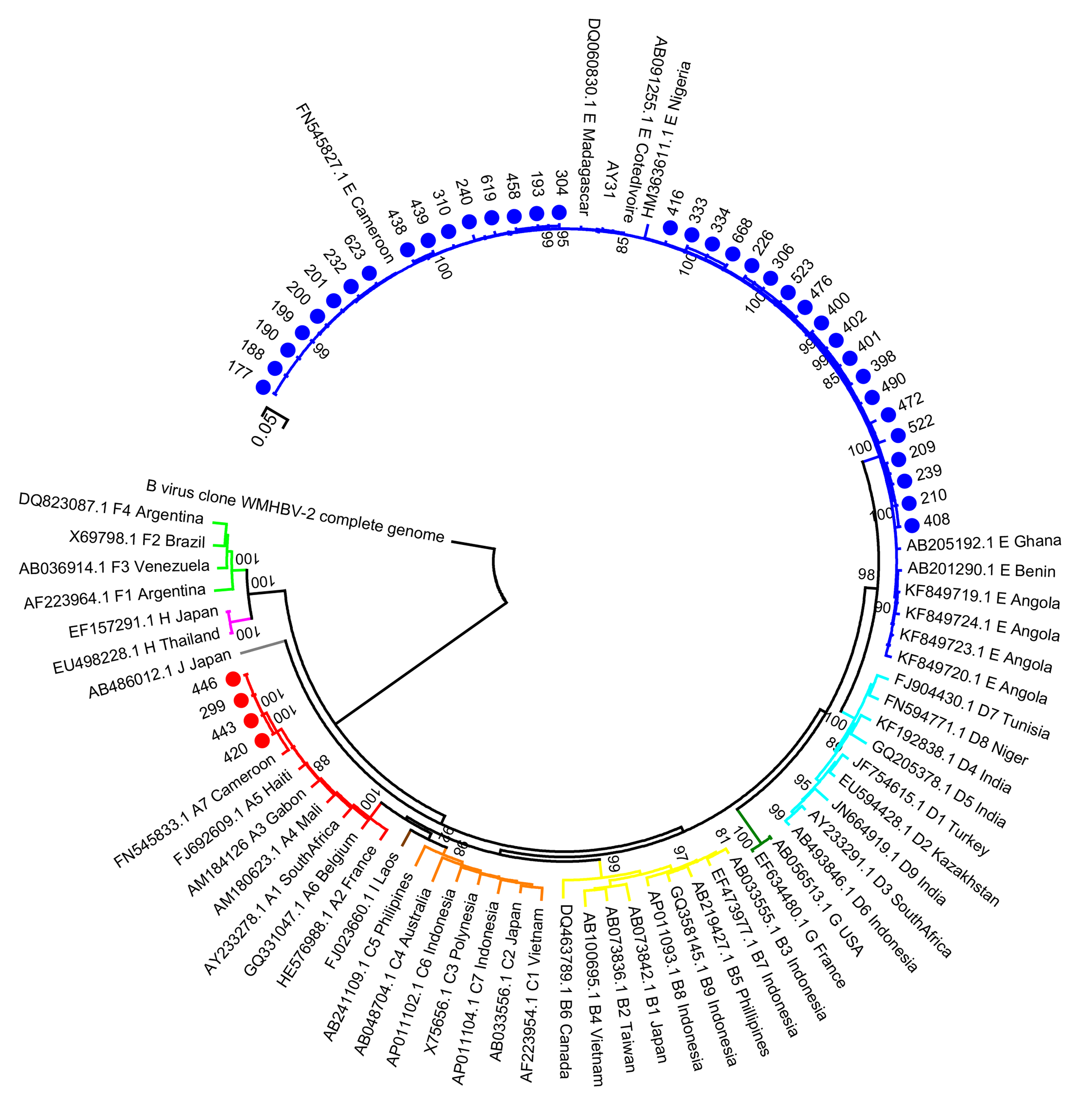

Supplement: FIG S1 [file sys005182262sf1.tif]

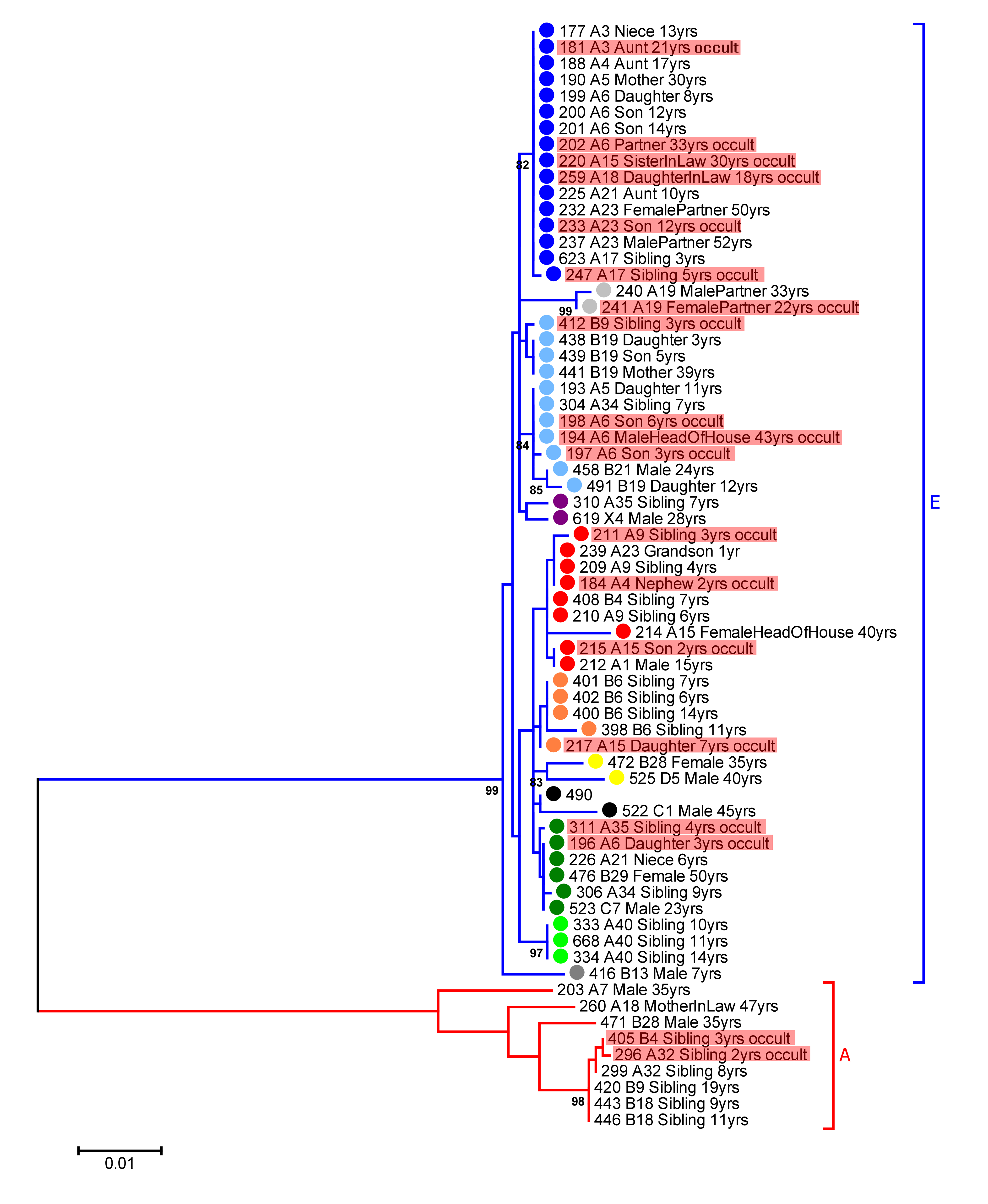

Supplement: FIG S2 [file sys005182262sf2.tif]

A

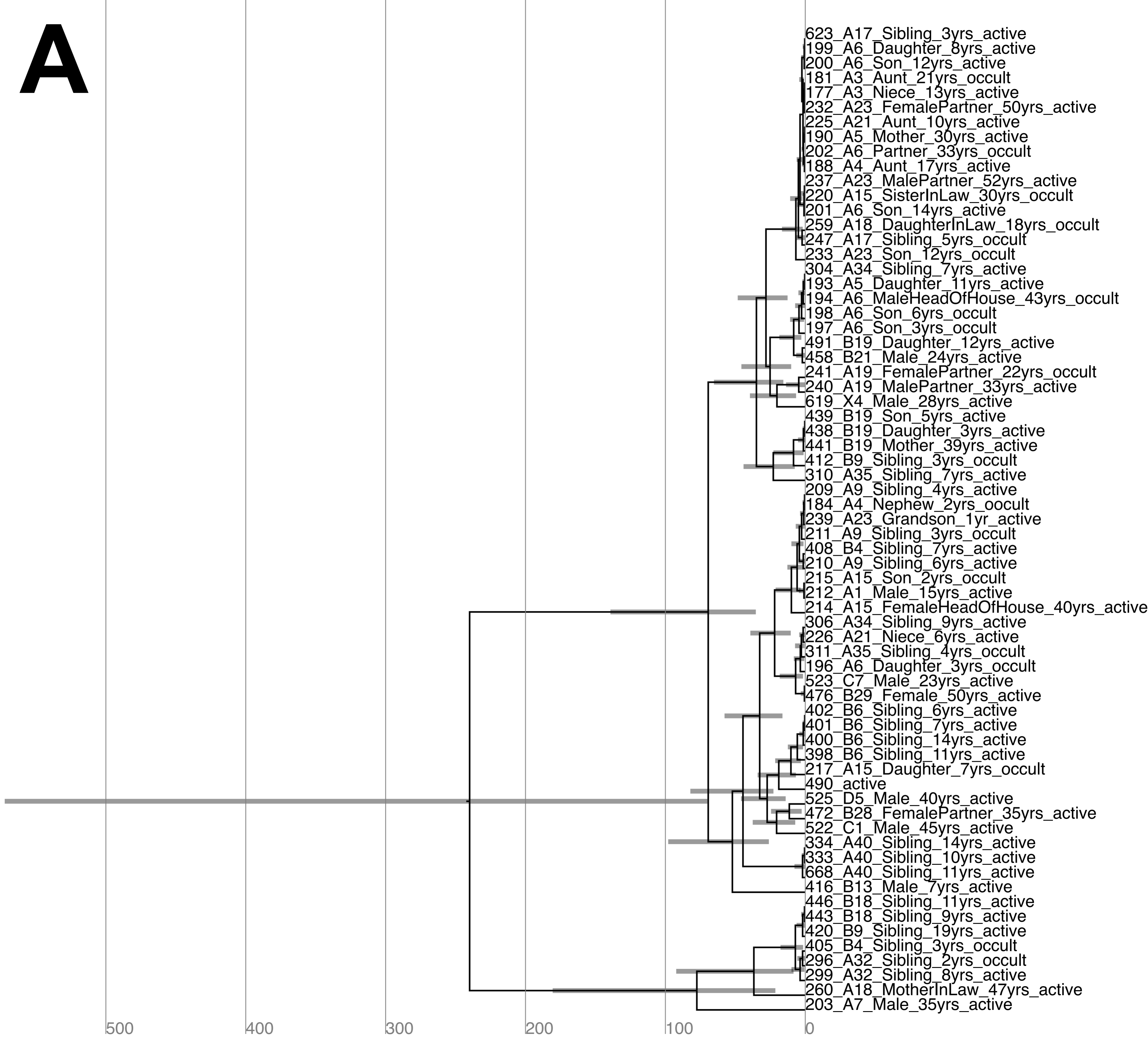

B

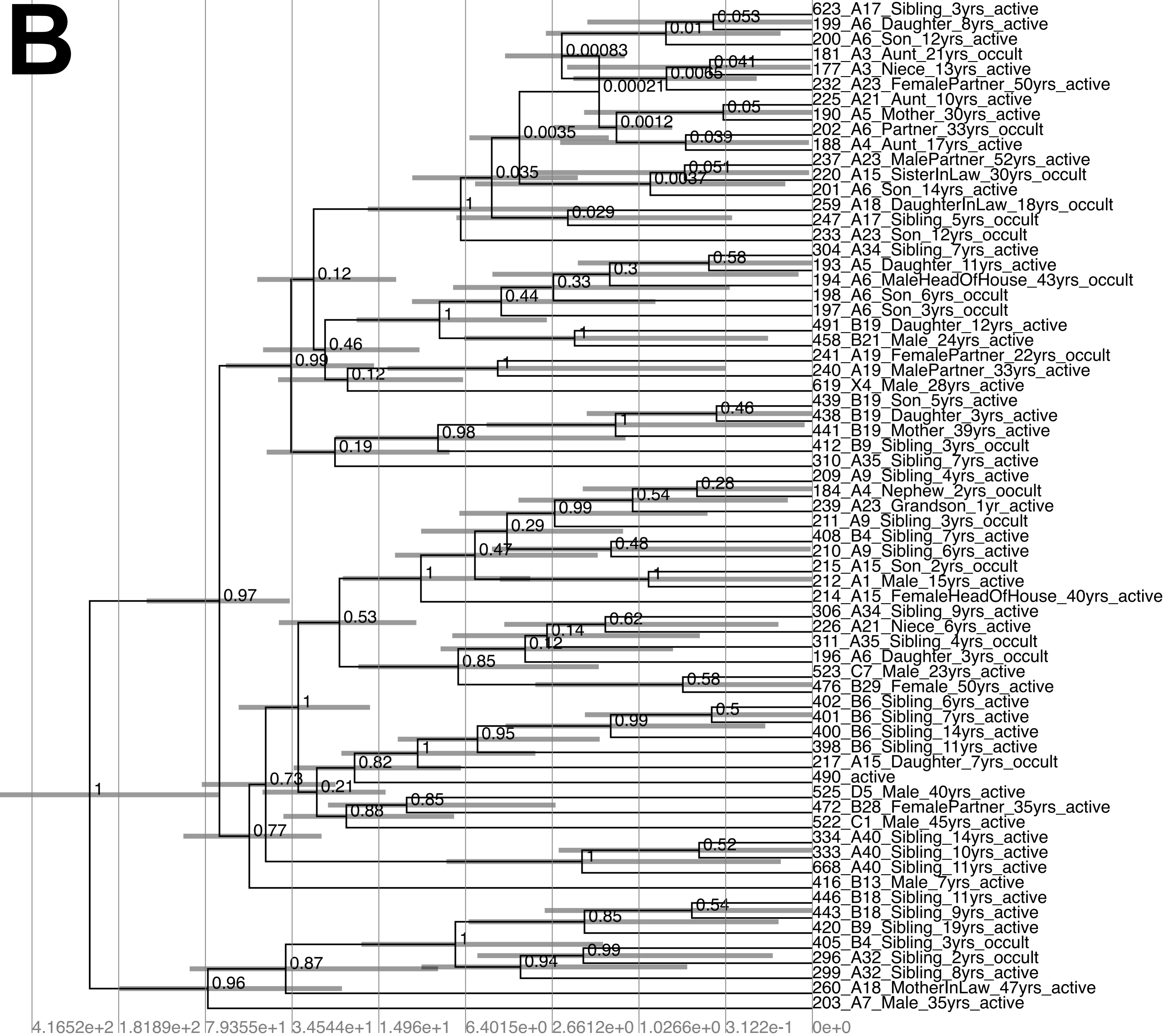

Supplement: FIG S3 [file sys005182262sf3.pdf]
