# Supplementary material for: Transmission of Hepatitis B and D Viruses in an African Rural Community
Source: mSystems. 2018 Sep 18;3(5):e00120-18. doi: 10.1128/mSystems.00120-18 (PMC6143728; doi:10.1128/mSystems.00120-18)
Supplement: TABLE S1 [file sys005182262st1.pdf]

Table S1. Primers used for HBV (whole genome and pre-S/S) and HDV (sHD gene fragment) nucleotide sequencing.

Sequencing target	Primer direction	Primer ID (Reference)	5'-Sequence-3'
HBV whole genome	Forward	C1 (1)	CTGTGGAGTTACTCTCGTTTTTGC
		P01 (2)	GGACTCATAAGGTGGGGAA
		P1(3)	CCGGAAAGCTTGAGCTCTTCTTTTTCACCTCTGCCTAATCA
		PS1 (1)	CCATATTCTTGGGAACAAGA
		PS4 (4)	ACACTCATCCTCAGGCCATGCAGTG
		S1 (2)	CTTCTCGAGGACTGGGGACC
		S4 (5)	TGCTGCTATGCCTCATCTTCT
		S18 (5)	GGATGATGTGGTATTGGGGGCCA
		X1 (1)	ACCTCCTTTCCATGGCTGCT
		X5 (2)	ACTCTTGGACTCBCAGCAATG
	Reverse	C8 (2)	GAGGGAGTTCTTCTTCTAGG
		P2 (3)	CCGGAAAGCTTGAGCTCTTCAAAAAGTTGCATGGTGCTGG
		P3 (2)	AAAGCCCAAAAGACCCACAA
		PS2 (1)	GGTCCCCAGTCCTCGAGAAG
		PS8 (4)	TTCCTGAACTGGAGCCACCA
		S2 (5)	GGGTTTAAATGTATACCCAAAGA
HBV PreS/S	Forward	PS1 (1)	CCATATTCTTGGGAACAAGA
		PS4 (4)	ACACTCATCCTCAGGCCATGCAGTG
	Reverse	PS8 (4)	TTCCTGAACTGGAGCCACCA
		S2 (5)	GGGTTTAAATGTATACCCAAAGA
HBV S	Forward	PS1a (4)	GGAAAACATCACATCAGGAT
		PS1b (4)	AAAATTCGCAGTCCCCAACC
	Reverse	P3 (2)	AAAGCCCAAAAGACCCACAA
HDV sHD gene fragment	Forward	HDV-E (6)	GAGATGCCATGCCGACCCGAAGAG
	Reverse	HDV-A (6)	GAAGGAAGGCCCTCGAGAACAAGA

Primer sequences were published previously by:

(1) Niel C, Moraes MT, Gaspar AM, Yoshida CF, Gomes SA. 1994. J Med Virol 44:180-6.
(2) de Pina-Araujo IIM, Spitz N, Soares CC, Niel C, Lago BV, Gomes SA. 2018. PLoS One 13:e0192595.
(3) Gunther S, Li BC, Miska S, Kruger DH, Meisel H, Will H. 1995. J Virol 69:5437-44.
(4) Ampah KA, Pinho-Nascimento CA, Kerber S, Asare P, De-Graft D, Adu-Nti F, Paixao IC, Niel C, Yeboah-Manu D, Pluschke G, Roltgen K. 2016. PLoS One 11:e0156864.
(5) Bottecchia M, Souto FJ, O KM, Amendola M, Brandao CE, Niel C, Gomes SA. 2008. BMC Microbiol 8:11.
(6) Casey JL, Brown TL, Colan EJ, Wignall FS, Gerin JL. 1993. Proc Natl Acad Sci U S A 90:9016-20.
