# Supplementary material for: Transmission of Hepatitis B and D Viruses in an African Rural Community
Source: mSystems. 2018 Sep 18;3(5):e00120-18. doi: 10.1128/mSystems.00120-18 (PMC6143728; doi:10.1128/mSystems.00120-18)
Supplement: TABLE S2 [file sys005182262st2.pdf]

Table S2. SNPs and variations in the predicted HBV/E and HBV/A amino acid sequences obtained in this study.

2a) Pre-S/S region of the 42 HBV/E sequences:

Nucleotide position	Amino acid position	Nucleotides	Amino acids	No. of sequences	Amino acid change	
6	2	G/A	G	39/2	No	Pre-S1
		G/T	G	39/1	No	
21	7	C/T	V	41/1	No	
27	9	C/A	L	39/3	No	
43	15	C/T	H/Y	39/3	Yes	
48	16	C/T	S	39/3	No	
76	26	G/A	D/N	41/1	Yes	
96	32	A/C	A	36/6	No	
100	34	A/C	R	41/1	No	
101	34	G/A	R/K	41/1	Yes	
112	38	A/C	R	41/1	No	
113	38	G/A	R/K	33/9	Yes	
117	39	T/C	N	40/2	No	
129	43	C/T	D	41/1	No	
157	53	G/A	E/K	40/1	Yes	
		G/C	E/Q	40/1	Yes*	
159	53	A/G	E/Q	41/1	Yes*	
198	66	C/T	F	41/1	No	
207	69	A/C	P	38/4	No	
225	75	G/A	G	41/1	No	
232	78	C/A	P/T	41/1	Yes	
254	85	A/C	K/T	41/1	Yes	
257	86	C/T	T/M	25/17	Yes	
258	86	A/G	T	21/21	No	
259	87	T/G	L/V	38/4	Yes	
265	89	G/A	A/T	32/10	Yes	
273	91	G/T	P	41/1	No	
285	95	C/A	S	41/1	No	
297	99	G/A	Q	41/1	No	
321	107	C/A	I	40/2	No	
326	109	C/T	P/L	40/2	Yes	
342	114	T/G	T	38/3	No	
		T/C	T	38/1	No	
350	117	A/T	Q/L	41/1	Yes	
355	119	A/G	M/V	41/1	Yes	Pre-S2
357	119	G/A	M/I	41/1	Yes	
359	120	A/G	Q/R	41/1	Yes	
360	120	G/A	Q	41/1	No	
370	124	A/C	T/P	41/1	Yes	
373	125	A/G	T/A	32/10	Yes	

385	129	G/A	A/T	41/1	Yes		
393	131	G/A	Q	41/1	No		
401	134	G/A	R/K	41/1	Yes		
407	136	G/A/(Del)	R/N/(Del)	40/1/(1)	Yes		
408	136	A/G/(Del)	R/(Del)	38/1/(2)	No		
418	140	T/C/(Del)	F/L/(Del)	29/1/(12)	Yes		
421	141	C/A	P/T	41/1	Yes		
440	147	C/T	S/F	41/1	Yes		
449	150	T/C	V/A	40/2	Yes		
466	156	A/T	T/S	41/1	Yes		
470	157	C/T	A/V	38/4	Yes		
509	170	C/T	P/L	41/1	Yes		
513	171	A/G	A	41/1	No		
515	172	C/G	P/R	38/4	Yes		
516	172	G/A	P	41/1	No		
527	176	G/A	S/N	41/1	Yes	S-region	
541	181	T/C	F/L	41/1	Yes		
555	185	G/A	L	41/1	No		
606	202	G/A	P	41/1	No		
607	203	C/A	Q/K	41/1	Yes		
652	218	G/A	A/T	41/1	Yes		
665	222	T/G	L/R	41/1	Yes		
678	226	G/A	S	41/1	No		
686	229	C/A	P/Q	41/1	Yes		
689	230	C/T	T/I	41/1	Yes		
692	231	C/G	S/C	41/1	Yes		
695	232	A/G	N/S	22/20	Yes		
722	241	T/C	I/T	41/1	Yes		
723	241	T/C	I	38/4	No		
732	244	C/T	G	39/3	No		
734	245	A/T	Y/F	41/1	Yes		
750	250	G/A	L	41/1	No		
765	255	C/A	I	39/3	No		
843	281	T/C	P	41/1	No		
853	285	G/A	G/R	41/1	Yes		
894	298	G/A	T	41/1	No		a-determinant
898	300	C/A	L	41/1	No		
899	300	T/C	L/P	41/1	Yes		
933	311	C/T	C	41/1	No		
938	313	C/T	S/L	41/1	Yes		
1032	344	C/T	S	41/1	No		
1085	362	C/T	T/I	40/2	Yes		
1127	376	C/A	P/Q	41/1	Yes		
1161	387	T/G	P	40/2	No		
1196	399	T/A	I/N	41/1	Yes		
1197	399	T/C	I	25/17	No		

2b) Pre-C/C region of the 35 HBV/E whole genome sequences:

Nucleotide position	Amino acid position	Nucleotides	Amino acids	No. of sequences	Amino acid change	Region
13	5	C/G	H/D	34/1	Yes	Pre-core
34	12	T/C	C/R	34/1	Yes	
83	28	G/A	W/Stop	33/2	Yes	
86	29	G/A	G/D	32/3	Yes	
96	32	T/C	I	34/1	No	Core
121	41	A/T	T/S	34/1	Yes	
125	42	T/A	V/E	34/1	Yes	
126	42	G/C	V	33/2	No	
138	46	G/A	S	32/3	No	
148	50	T/C	S/P	34/1	Yes	
156	52	C/T	F	31/4	No	
170	57	G/A	R/K	34/1	Yes	
186	62	C/T	T	34/1	No	
187	63	G/A	A	34/1	No	
188	63	C/T	A/I	34/1	Yes	
200	67	A/T	Y/F	33/2	Yes	
220	74	C/T	P/S	34/1	Yes	
231	77	T/C	C	34/1	No	
240	80	C/T	H	34/1	No	
243	81	T/C	H	19/16	No	
249	83	A/T	A	33/2	No	
250	84	C/A	L/I	34/1	Yes	
276	92	G/A	G	34/1	No	
277	93	G/A	E/N	34/1	Yes*	
279	93	A/C	E/N	32/1	Yes*	
		A/C	E/D	32/1	Yes	
		A/T	E/D	32/1	Yes	
280	94	C/T	L	34/1	No	
282	94	A/T	L	34/1	No	
287	96	C/A	T/N	33/2	Yes	
291	97	A/G	L	34/1	No	
293	98	C/G	A/G	34/1	Yes*	
294	98	T/C	A	29/5	No	
		T/A	A/G	29/1	Yes*	
306	102	T/G	G	32/3	No	
308	103	T/G	V/G	33/1	Yes	
		T/C	V/A	33/1	Yes	
312	104	T/C	N	34/1	No	
323	108	C/A	P/Q	34/1	Yes	
326	109	C/G	A/G	34/1	Yes	
336	112	C/A	D/E	34/1	Yes	

345	115	T/C	V	19/16	No
354	118	C/T	V	31/4	No
361	121	A/C	N/H	34/1	Yes
363	121	T/C	N	34/1	No
369	123	C/A	G	31/4	No
373	125	A/C	K/Q	34/1	Yes
381	127	G/A	R	30/5	No
388	130	T/A	L/M	34/1	Yes
402	134	T/G	I/M	34/1	Yes
409	137	C/A	L/I	34/1	Yes
411	137	C/T	L	31/4	No
413	138	C/T	T/I	34/1	Yes
420	140	A/G	G	34/1	No
423	141	A/G	R	34/1	No
427	143	A/C	T/L	34/1	Yes*
428	143	C/T	T/L	34/1	Yes*
429	143	C/T	T	20/15	No
433	145	A/G	I/V	33/1	Yes
		A/T	I/L	33/1	Yes
447	149	G/A	V	34/1	No
476	159	C/A	P/Q	33/2	Yes
477	159	A/C	P	34/1	No
483	161	C/T	Y	18/17	No
489	163	A/G	P	34/1	No
491	164	C/A	P/Q	34/1	Yes
519	173	G/T	P	34/1	No
524	175	A/C	N/T	31/4	Yes
526	176	A/T	T/S	33/1	Yes
		A/G	T/A	33/1	Yes
538	180	C/T	R/S	34/1	Yes*
539	180	G/C	R/S	34/1	Yes*
541	181	A/C	R	32/3	No
553	185	C/T	P/S	34/1	Yes
610	204	A/C	R	34/1	No
625	209	G/T	A/S	34/1	Yes
628	210	T/G	S/A	32/2	Yes
		T/C	S/P	32/1	Yes
632	211	A/G	Q/R	34/1	Yes

2c) X region of the 35 HBV/E whole genome sequences:

Nucleotide position	Amino acid position	Nucleotides	Amino acids	No. of sequences	Amino acid change
34	12	G/T	A/S	34/1	Yes
37	13	A/C	R	34/1	No
64	22	A/G	S/G	32/3	Yes
65	22	G/A	S/N	34/1	Yes
73	25	T/C	S/P	32/3	Yes
88	30	G/A	V/I	33/2	Yes
101	34	T/C	L/P	31/4	Yes
103	35	G/A	G/R	33/2	Yes
107	36	A/G	D/G	24/11	Yes
109	37	C/T	L	32/3	No
111	37	A/G	L	26/9	No
127	43	T/A	S/T	27/8	Yes
138	46	A/G	P	31/4	No
143	48	A/T	D/V	31/4	Yes
154	52	C/A	H/N	34/1	Yes
192	64	A/G	S	32/3	No
201	67	A/T	G	28/7	No
217	73	T/G	F/V	34/1	Yes
240	80	G/A	E	34/1	No
260	87	A/G	Q/R	34/1	Yes
262	88	A/C	I/L	19/16	Yes*
264	88	C/A	I/L	31/2	Yes*
		C/A	I	31/1	No
		C/T	I	31/1	No
280	94	C/T	H/Y	34/1	Yes
340	114	G/A	D/N	31/4	Yes
351	117	T/G	F/L	33/1	Yes
		T/C	F	33/1	No
354	118	G/A	K	32/3	No
366	122	G/A	E	34/1	No
368	123	T/C	L/S	18/17	Yes
384	128	A/G	R	30/5	No
387	129	A/G	L	34/1	No
389	130	A/T	K/M	33/2	Yes
391	131	G/A	V/I	33/2	Yes
436	146	G/T	A/S	34/1	Yes
453	151	C/G	F/L	34/1	Yes

2d) P region of the 35 HBV/E whole genome sequences:

Nucleotide position	Amino acid position	Nucleotides	Amino acids	No. of sequences	Amino acid change
26	9	G/T	R/L	34/1	Yes
31	11	A/C	I/L	31/4	Yes*
33	11	A/T	I/L	33/1	Yes*
		A/G	I/L	33/1	Yes*
45	15	C/T	D	34/1	No
46	16	G/C	E/Q	34/1	Yes
48	16	A/C	E/D	32/3	Yes
60	20	C/T	P	34/1	No
117	39	A/C	E/D	34/1	Yes
132	44	G/T	Q/H	34/1	Yes
135	45	T/G	L	32/2	No
		T/C	L	32/1	No
139	47	A/G	N/D	34/1	Yes
153	51	T/G	P	34/1	No
171	57	A/C	G	34/1	No
199	67	A/T	I/L	31/4	Yes
200	67	T/C	I/T	34/1	Yes
201	67	A/T	I	34/1	No
205	69	G/A	V/I	31/4	Yes
228	76	T/A	T	32/3	No
240	80	T/C	P	34/1	No
252	84	G/A	L	34/1	No
267	89	T/C	I	34/1	No
268	90	A/C	N/H	34/1	Yes
270	90	C/T	N	31/4	No
291	97	T/C	G	32/3	No
294	98	T/G	P	33/2	No
295	99	C/T	L	34/1	No
297	99	A/G	L	34/1	No
303	101	A/T	V	32/3	No
313	105	C/A	R	32/3	No
316	106	A/C	R	32/3	No
318	106	A/G	R	34/1	No
324	108	C/A	N/K	34/1	Yes
330	110	C/T	V	30/5	No
331	111	A/C	M/L	34/1	Yes
360	120	G/A	T	29/6	No
369	123	A/G	L	19/15	No
		A/T	L/F	19/1	Yes
372	124	C/G	P	32/2	No
		C/T	P	32/1	No

375	125	A/G	L	34/1	No
381	127	A/G	K	32/3	No
387	129	A/C	I	33/2	No
393	131	T/G	P	34/1	No
402	134	A/C	P	34/1	No
411	137	A/G	V	27/8	No
426	142	C/T	F	34/1	No
433	145	A/C	R	33/2	No
450	150	C/T	T	34/1	No
453	151	A/T	L	34/1	No
462	154	G/A	A	34/1	No
465	155	C/T	G	27/8	No
480	160	A/G	R	21/14	No
486	162	T/G	T	20/15	No
506	169	T/G	F/C	19/16	Yes
534	178	G/C	E/D	34/1	Yes
547	183	G/A	A/T	33/2	Yes
562	188	C/T	P/S	34/1	Yes
568	190	C/A	R	32/3	No
584	195	C/T	S/L	32/3	Yes
589	197	C/T	H/Y	33/2	Yes
637	213	A/C	I/L	31/4	Yes
641	214	A/C	Q/P	34/1	Yes
642	214	G/A	Q	34/1	No
653	218	A/C	Q/P	34/1	Yes
654	218	G/A	Q	28/7	No
658	220	T/C	S/P	34/1	Yes
698	233	G/C	R/T	34/1	Yes
700	234	A/G	S/G	34/1	Yes
739	247	C/T	H/Y	34/1	Yes
748	250	A/C	T/P	31/4	Yes
773	258	C/A	P/H	34/1	Yes
795	265	A/C	K/N	34/1	Yes
798	266	C/T	N	21/14	No
799	267	G/A	V/I	18/17	Yes
800	267	T/G	V/S	32/3	Yes
806	269	G/A	S/N	27/8	Yes
814	272	G/T	A/S	34/1	Yes
838	280	G/A	V/I	34/1	Yes
862	288	C/A	H/N	33/2	Yes
867	289	C/T	S	34/1	No
883	295	T/G	S/A	32/2	Yes
		T/C	S/P	32/1	Yes
891	297	A/T	S	34/1	No

896	299	A/G	H/R	34/1	Yes
900	300	A/G	A	34/1	No
911	304	A/C	H/P	34/1	Yes
914	305	A/G	N/S	27/8	Yes
926	309	G/A	S/N	34/1	Yes
934	312	G/A	G/R	34/1	Yes
942	314	G/A	Q	34/1	No
948	316	G/A/(Del)	K/(Del)	33/1/(1)	No
981	327	C/T	F	34/1	No
990	330	C/T	S	34/1	No
1007	336	A/T	Y/F	34/1	Yes
1011	337	C/T	C	32/3	No
1050	350	C/T	P	34/1	No
1054	352	A/G	T/A	34/1	Yes
1056	352	C/G	T	31/4	No
1148	383	C/A	A/E	34/1	Yes
1206	402	T/G	S	34/1	No
1227	409	C/A	P	34/1	No
1230	410	C/T	N	34/1	No
1233	411	C/G	L	34/1	No
1236	412	A/G	Q	19/16	No
1263	421	T/C	N	34/1	No
1264	422	T/C	L	31/4	No
1273	425	C/T	L	32/3	No
1275	425	A/T	L	34/1	No
1306	436	C/A	L/I	33/2	Yes
1384	462	T/C	S/P	34/1	Yes
1394	465	G/A	R/K	34/1	Yes
1435	479	G/A	D/N	34/1	Yes
1439	480	C/A	S/Y	34/1	Yes
1440	480	T/C	S	34/1	No
1474	492	C/T	L	34/1	No
1479	493	C/T	F	34/1	No
1573	525	C/T	L	34/1	No
1626	542	C/T	H	34/1	No
1668	556	C/A	A	34/1	No
1702	568	T/G	S/A	33/2	Yes
1737	579	T/A	H/Q	34/1	Yes
1738	580	T/C	L	20/15	No
1744	582	C/T	P/S	31/3	Yes
		C/G	P/A	31/1	Yes
1746	582	C/T	P	34/1	No
1749	583	C/T	N	34/1	No
1759	587	A/C	R	31/4	No

1761	587	A/G	R	34/1	No
1773	591	C/A	S	34/1	No
1774	592	C/T	L	34/1	No
1777	593	A/C	N/H	20/15	Yes
1788	596	T/C	G	32/3	No
1805	602	G/T	W/F	34/1	Yes
1806	602	G/T	W	34/1	No
1809	603	G/A	G	32/2	No
		G/C	G	32/1	No
1812	604	A/T	S	33/2	No
1821	607	G/A	Q	31/4	No
1830	610	C/T	I	34/1	No
1832	611	G/A	R/K	18/17	Yes
1834	612	A/T	L/M	19/16	Yes
1836	612	G/A	L	26/9	No
1872	624	C/T	N	34/1	No
1875	625	G/A	R	32/3	No
1903	635	A/C	I/L	32/3	Yes
1920	640	C/T	G	32/3	No
1944	648	T/C	C	34/1	No
1950	650	T/C	Y	34/1	No
1965	655	T/G	P	34/1	No
1968	656	G/A	L	34/1	No
1972	658	G/A	A/T	30/5	Yes
1974	658	G/A	A	34/1	No
1979	660	T/C	I/T	24/11	Yes
1984	662	T/G	S/A	31/4	Yes
1985	662	C/A	S/Y	31/4	Yes
2004	668	C/T	F	34/1	No
2022	674	C/T	A	34/1	No
2032	678	A/C	K/Q	30/5	Yes
2041	681	C/A	L/M	34/1	Yes
2042	681	T/A	L/Q	33/1	Yes
		T/G	L/R	33/1	Yes
2044	682	A/T	N/Y	34/1	Yes
2071	691	C/T	P/S	34/1	Yes
2072	691	C/A	P/Q	31/4	Yes
2073	691	A/T	P	34/1	No
2088	696	G/A	V	32/3	No
2112	704	C/T	G	32/3	No
2127	709	A/G	I/M	34/1	Yes
2139	713	C/T	R	34/1	No
2140	714	A/G	M/V	32/3	Yes
2212	738	A/C	R	31/4	No

2220	740	A/G	G	27/8	No
2223	741	G/A	A	20/14	No
		G/T	A	20/1	No
2224	742	A/C	K/Q	34/1	Yes
2225	742	A/C	K/T	30/5	Yes
2232	744	C/T	I	33/2	No
2235	745	G/A	G	27/8	No
2238	746	T/A	T	18/17	No
2247	749	T/C	S	31/4	No
2250	750	C/T	V	32/3	No
2274	758	A/C	S	34/1	No
2313	771	G/T	L	34/1	No
2316	772	A/C	R	34/1	No
2343	781	A/G	S	32/3	No
2344	782	G/A	A/T	34/1	Yes
2352	784	T/C	N	32/3	No
2367	789	G/A	P	33/2	No
2380	794	T/C	L	31/4	No
2382	794	G/A	L	33/2	No
2386	796	A/G	I/V	24/11	Yes
2388	796	C/T	I	32/3	No
2390	797	A/G	Y/C	26/9	Yes
2406	802	T/A	R	27/8	No
2417	806	A/G	Q/R	31/4	Yes
2422	808	A/T	T/S	31/4	Yes
2433	811	C/A	R	34/1	No
2471	824	A/G	H/R	32/3	Yes
2480	827	A/T	D/V	28/7	Yes
2496	832	T/G	A	34/1	No

2e) Pre-S/S region of the 7 HBV/A sequences:

Nucleotide position	Amino acid position	Nucleotides	Amino acids	No. of sequences	Amino acid change	Region	
6	2	A/G	G	6/1	No	Pre-S1	
13	5	T/A	L/I	6/1	Yes		
96	32	T/C	P	6/1	No		
159	53	G/A	P	4/3	No		
183	61	A/C	G	6/1	No		
217	73	G/A	G/S	6/1	Yes		
233	78	G/C	S/T	6/1	Yes		
289	97	A/G	T/A	6/1	Yes		
305	102	G/A	G/E	6/1	Yes		
318	106	T/A	T	6/1	No		
342	114	G/C	E/D	4/3	Yes		
360	120	G/A	M/I	6/1	Yes	Pre-S2	
363	121	G/A	Q	6/1	No		
376	126	A/C	T/P	6/1	Yes		
384	128	C/T	H	5/2	No		
390	130	T/C	A	6/1	No		
393	131	G/A	L	6/1	No		
405	135	G/A	R	6/1	No		
412	138	G/A	G/S	6/1	Yes		
414	138	T/C	G	4/3	No		
421	141	T/C	F/L	6/1	Yes		
423	141	C/T	F	6/1	No		
428	143	C/T	A/V	5/2	Yes		
440	147	A/G	N/S	6/1	Yes		
461	154	T/C	V/A	6/1	Yes		
467	156	C/A	T/N	4/3	Yes		
468	156	C/T	T/N	4/3	Yes		
470	157	C/T	T/I	6/1	Yes		
486	162	A/G	S	6/1	No		
533	178	T/C	I/T	6/1	Yes	S-region	
609	203	G/A	P	5/2	No		
655	219	T/A	S/T	6/1	Yes		
813	271	G/T	L	6/1	No		
930	310	A/C	S	6/1	No		a
939	313	T/C	C	6/1	No		
1103	368	T/C	V/A	6/1	Yes		
1121	374	A/T	Y/F	6/1	Yes		
1140	380	C/T	Y	6/1	No		
1141	381	A/C	S/R	6/1	Yes		
1142	381	G/A	S/N	5/2	Yes		
1172	391	C/T	P/L	6/1	Yes		

1188	396	T/C	L	6/1	No	
1193	398	C/T	A/V	5/2	Yes	
