# Supplementary material for: Transmission of Hepatitis B and D Viruses in an African Rural Community
Source: mSystems. 2018 Sep 18;3(5):e00120-18. doi: 10.1128/mSystems.00120-18 (PMC6143728; doi:10.1128/mSystems.00120-18)
Supplement: TABLE S3 [file sys005182262st3.pdf]

Table S3. Replacements in predicted HBV/E and HBV/A amino acid sequences obtained in this study.

3a) Pre-S/S region of the 42 HBV/E sequences as well as the 17 OBI HBV/E sequences:

	Pre-S1													Pre-S2																		S-region																		
Site	14	15	26	34	38	53	78	85	86	87	89	109	117	1	2	6	7	11	16	18	19	20	21	22	23	29	32	38	39	52	54	3	8	10	30	45	49	56	57	58	59	68	72	112	127	140	165	189	203	226
Consensus	N	H	D	R	R	E	P	K	T	L	A	P	Q	M	Q	T	T	A	R	R	G	L	Y	F	P	S	V	T	A	P	P	S	F	G	Q	A	L	P	T	S	S	I	Y	G	L	S	W	T	P	I
177	.	.	.	.	.	.	.	.	.	.	T	.	.	.	.	.	A	.	.	.	.	.	.	---	.	.	.	.	.	.	.	.	.	.	.	.	.	.	.	.	.	.	.	.	.	.	.	.	.	.
188	.	.	.	.	.	.	.	.	.	.	T	.	.	.	.	.	A	.	.	.	.	.	.	---	.	.	.	.	.	.	.	.	.	.	.	.	.	.	.	.	.	.	.	.	.	.	.	.	.	.
190	.	.	.	.	.	.	.	.	.	.	T	.	.	.	.	.	A	.	.	.	.	.	.	---	.	.	.	.	.	.	.	.	.	.	.	.	.	.	.	.	.	.	.	.	.	.	.	.	.	.
193	.	.	.	.	.	.	.	.	.	V	.	.	.	.	.	.	.	.	.	.	.	.	.	.	.	.	.	.	V	.	.	.	.	.	.	.	.	.	.	.	.	.	.	.	.	.	.	.	.	.
199	.	.	.	.	.	.	.	.	.	.	T	.	.	.	.	.	A	.	.	.	.	.	.	---	.	.	.	.	.	.	.	.	.	.	.	.	.	.	.	.	.	.	.	.	.	.	.	.	.	.
200	.	.	.	.	.	.	.	.	.	.	T	.	.	.	.	.	A	.	.	.	.	.	.	---	.	.	.	.	.	.	.	.	.	.	.	.	.	.	.	.	.	.	.	.	.	.	.	.	.	.
201	.	.	.	.	.	.	.	.	.	.	T	.	.	.	.	.	A	.	.	.	.	.	.	---	.	.	.	.	.	.	.	.	.	.	.	.	.	.	.	.	.	.	.	.	.	.	.	.	.	.
209	.	.	.	.	K	.	.	.	M	.	.	.	.	.	.	.	.	.	.	.	.	.	.	.	.	.	.	.	.	.	.	.	.	.	.	.	.	.	.	.	N	.	.	.	.	.	.	.	.	.
210	.	.	.	.	K	.	.	.	M	.	.	.	.	.	.	.	.	.	.	.	.	.	.	.	.	.	.	.	.	.	.	.	.	.	.	.	.	.	.	.	N	.	.	.	.	.	.	.	.	.
212	.	.	.	.	K	.	.	.	M	.	.	.	.	.	.	.	.	.	.	.	.	.	.	.	.	.	.	.	.	.	.	.	.	.	.	.	.	.	.	.	N	.	.	.	.	.	.	.	.	.
214	.	.	N	.	K	K	.	.	M	.	.	.	.	I	.	.	.	.	.	.	.	.	.	.	.	.	.	.	.	.	.	N	.	.	.	.	.	.	.	.	N	.	.	.	.	.	.	.	.	.
225	.	.	.	.	.	.	.	.	.	.	T	.	.	.	.	.	A	.	.	.	.	.	.	---	.	.	.	.	.	.	.	.	.	.	.	.	.	.	.	.	.	.	.	.	.	.	.	.	.	.
226	.	.	.	.	.	.	.	.	M	.	.	.	.	.	.	.	.	.	.	.	.	.	.	.	.	.	.	.	.	.	.	.	.	.	.	.	.	.	.	.	N	.	.	.	.	.	.	.	.	.
232	.	.	.	.	.	.	.	.	.	.	T	.	.	.	.	.	A	.	.	.	.	.	.	---	.	.	.	.	.	.	.	.	.	.	.	.	.	.	.	.	.	.	.	.	.	.	.	.	.	.
237	.	.	.	.	.	.	.	.	.	.	T	.	.	.	.	.	A	.	.	.	.	.	.	---	.	.	.	.	.	.	.	.	.	.	.	.	.	.	.	.	.	.	.	.	.	.	.	.	.	.
239	.	.	.	.	K	.	.	.	M	.	.	.	.	.	.	.	.	.	.	.	.	.	.	.	.	.	.	.	.	.	.	.	.	.	.	.	.	.	.	.	N	.	.	.	.	.	.	.	.	.
240	.	.	.	.	.	.	T	T	.	.	.	.	.	.	.	.	.	.	K	.	---	---	---	---	.	F	.	.	.	L	.	.	.	.	K	.	.	.	.	.	.	.	.	.	.	L	.	.	Q	.
304	.	.	.	.	.	.	.	.	.	V	.	.	.	.	.	.	.	.	.	.	.	.	.	.	.	.	.	.	V	.	.	.	.	.	.	.	.	.	.	.	.	.	.	.	.	.	.	.	.	.
306	.	.	.	.	.	.	.	.	M	.	.	.	.	.	.	.	.	.	.	.	.	.	.	.	.	.	.	S	.	.	.	.	.	.	.	.	.	.	.	.	N	.	.	.	.	.	.	.	.	.
310	.	.	.	.	.	.	.	.	.	.	.	.	.	.	.	.	.	T	.	.	.	.	.	.	.	.	.	.	.	.	.	.	.	.	.	.	.	.	.	.	.	.	.	.	.	.	.	.	.	.
333	.	Y	.	.	K	.	.	.	.	.	.	.	.	.	.	.	.	.	.	.	.	.	.	.	.	.	.	.	.	.	.	.	.	.	.	.	.	.	.	.	N	.	.	.	.	.	.	.	.	.
334	.	Y	.	.	K	.	.	.	.	.	.	.	.	.	.	.	.	.	.	.	.	.	.	.	.	.	.	.	.	.	.	.	.	.	.	.	.	.	.	.	N	.	.	.	.	.	.	.	.	.
398	.	.	.	.	.	.	.	.	.	.	.	.	.	.	.	.	.	.	.	---	---	---	---	---	.	.	.	.	.	.	R	.	.	.	.	.	R	.	.	.	N	.	.	.	I	.	.	I	.	.
400	.	.	.	.	.	.	.	.	M	.	.	.	.	.	.	.	.	.	.	.	.	.	.	.	.	.	.	.	.	.	R	.	.	.	.	.	.	.	.	.	N	.	.	.	.	.	.	.	.	.
401	.	.	.	.	.	.	.	.	M	.	.	.	.	.	.	.	.	.	.	.	.	.	.	.	.	.	.	.	.	.	R	.	.	.	.	.	.	.	.	.	N	.	.	.	.	.	.	.	.	.
402	.	.	.	.	.	.	.	.	M	.	.	.	.	.	.	.	.	.	.	.	.	.	.	.	.	.	.	.	.	.	R	.	.	.	.	.	.	.	.	.	N	.	.	.	.	.	.	.	.	.
408	.	.	.	.	K	.	.	.	M	.	.	.	.	.	.	.	.	.	.	.	.	.	.	.	.	.	.	.	.	.	.	.	.	.	.	.	.	.	.	.	N	.	.	.	.	.	.	.	.	.
416	.	.	.	.	.	.	.	.	.	.	.	.	.	.	.	.	.	.	.	.	.	.	.	.	.	.	.	.	.	.	.	.	.	.	.	.	.	Q	I	.	N	.	.	.	P	.	.	.	.	N
438	.	.	.	.	.	.	.	.	.	.	.	.	.	.	.	.	.	.	.	.	.	.	.	.	.	.	.	.	.	.	.	.	.	.	.	.	.	.	.	.	.	.	.	.	.	.	.	.	.	.
439	.	.	.	.	.	.	.	.	.	.	.	.	.	.	.	.	.	.	.	.	.	.	.	.	.	.	.	.	.	.	.	.	.	.	.	.	.	.	.	.	.	.	.	.	.	.	.	.	.	.
441	.	.	.	.	.	.	.	.	.	.	.	.	.	.	.	.	.	.	.	.	.	.	.	.	.	.	.	.	.	.	.	.	.	.	.	.	.	.	.	.	.	.	.	.	.	.	.	.	.	.
491	.	.	.	.	.	.	.	.	.	V	.	L	.	.	.	.	.	.	.	.	.	.	.	.	.	.	A	.	V	.	.	.	.	.	.	.	.	.	.	.	.	.	.	.	.	.	.	I	.	.
458	.	.	.	.	.	.	.	.	.	V	.	L	.	.	.	.	.	.	.	.	.	.	.	.	.	.	A	.	V	.	.	.	.	.	.	.	.	.	.	.	.	.	.	.	.	.	.	.	.	.
472	.	.	.	.	.	.	.	.	M	.	.	.	.	.	.	.	.	.	.	.	.	.	.	.	.	.	.	.	.	.	.	.	.	.	.	.	.	.	.	C	N	T	F	R	.	.	.	.	.	.
476	.	.	.	.	.	.	.	.	M	.	.	.	.	.	.	.	.	.	.	.	.	.	.	.	.	.	.	.	.	.	.	.	.	.	.	.	.	.	.	.	N	.	.	.	.	.	.	.	.	.
490	.	.	.	.	.	.	.	.	M	.	.	.	.	.	.	.	.	.	.	.	.	.	.	.	.	.	.	.	.	.	.	.	.	.	.	.	.	.	.	.	N	.	.	.	.	.	.	.	.	.
522	.	.	.	K	.	.	.	.	M	.	.	.	L	V	.	.	.	.	.	.	.	.	.	.	.	.	.	.	.	.	.	.	.	.	.	.	.	.	.	.	N	.	.	.	.	.	.	.	.	.
523	.	.	.	.	.	.	.	.	M	.	.	.	.	.	.	.	.	.	.	.	.	.	.	.	.	.	.	.	.	.	.	.	.	.	.	.	.	.	.	.	N	.	.	.	.	.	.	.	.	.
525	.	.	.	.	.	.	.	.	M	.	.	.	.	.	.	.	.	.	.	.	.	.	.	L	.	.	.	.	.	.	.	.	L	.	.	T	.	.	.	.	N	.	.	.	.	.	.	.	.	.
619	.	.	.	.	.	.	.	.	.	.	.	.	.	.	R	P	.	.	.	.	.	.	.	.	.	.	.	.	.	.	.	.	.	.	.	.	.	.	.	.	.	.	.	.	.	.	.	.	.	.

623	.	.	.	.	.	.	.	.	.	.	T	.	.	.	.	.	A	.	.	.	.	.	.	---	.	.	.	.	.	.	.	.	.	.	.	.	.	.	.	.	.	.	.	.	.	.	.	.	.	.
668	.	Y	.	.	K	.	.	.	.	.	.	.	.	.	.	.	.	.	.	.	.	.	.	.	.	.	.	.	.	.	.	.	.	.	.	.	.	.	.	.	N	.	.	.	.	.	.	.	.	.
181occult	.	.	.	.	.	.	.	.	.	.	T	.	.	.	.	.	A	.	.	.	.	.	.	---	.	.	.	.	.	.	.			.	.	.	.	.	.	.	.	.	.	.	.	.	.	.	.	.
184occult	.	.	.	.	K	.	.	.	M	.	.	.	.	.	.	.	.	.	.	.	.	.	.	.	.	.	.	.	.	.	.			.	.	.	.	.	.	.	N	.	.	.	.	.	.	.	.	.
194occult	.	.	.	.	.	.	.	.	.	V	.	.	.	.	.	.	.	.	.	.	.	.	.	.	.	.	.	.	V	.	.			.	.	.	.	.	.	.	.	.	.	.	.	.	.	.	.	.
196occult	.	.	.	.	.	.	.	.	M	.	.	.	.	.	.	.	.	.	.	.	.	.	.	.	.	.	.	.	.	.	.			.	.	.	.	.	.	.	N	.	.	.	.	.	.	.	.	.
197occult	D	.	.	.	.	.	.	.	.	V	.	.	.	.	.	.	.	.	.	.	.	.	.	.	.	.	.	.	V	.	.			.	.	.	.	.	.	.	.	.	.	.	.	.	.	.	.	.
198occult	.	.	.	.	.	.	.	.	.	V	.	.	.	.	.	.	.	.	.	.	.	.	.	.	.	.	.	.	V	.	.			.	.	.	.	.	.	.	.	.	.	.	.	.	.	.	.	.
202occult	.	.	.	.	.	.	.	.	.	.	T	.	.	.	.	.	A	.	.	.	.	.	.	---	.	.	.	.	.	.	.			.	.	.	.	.	.	.	.	.	.	.	.	.	.	.	.	.
211occult	.	.	.	.	K	.	.	.	M	.	.	.	.	.	.	.	.	.	.	.	.	.	.	.	.	.	.	.	.	.	.		.	R	.	.	.	.	.	.	N	.	.	.	.	.	R	.	.	.
215occult	.	.	.	.	K	.	.	.	M	.	.	.	.	.	.	.	.	.	.	.	.	.	.	.	.	.	.	.	.	.	.		.	.	.	.	.	.	.	.	N	.	.	.	.	.	.	.	.	.
217occult	.	.	.	.	.	.	.	.	M	.	.	.	.	.	.	.	.	.	.	.	.	.	.	.	.	.	.	.	.	.	R		.	.	.	.	.	.	.	.	N	.	.	.	.	.	.	.	.	.
220occult	.	.	.	.	.	.	.	.	.	.	T	.	.	.	.	.	A	.	.	.	.	.	.	---	.	.	.	.	.	.	.		.	.	.	.	.	.	.	.	.	.	.	.	.	.	.	.	.	.
233occult	.	.	.	.	.	.	.	.	.	.	T	.	.	.	.	.	A	.	.	.	.	.	.	---	.	.	.	.	.	.	.		.	.	.	.	.	.	.	.	.	.	.	.	.	.	.	.	.	.
241occult	.	.	.	.	.	.	T	T	.	.	.	.	.	.	.	.	.	.	.	.	---	---	---	---	.	F	.	.	.	L	.		.	.	K	.	.	.	.	.	.	.	.	.	.	L	.	.	.	.
247occult	.	.	.	.	.	.	.	.	.	.	T	.	.	.	.	.	A	.	.	.	.	.	.	---	.	.	.	.	.	.	.		.	.	.	.	.	.	.	.	.	.	.	.	.	.	.	.	.	.
259occult	.	.	.	.	.	.	.	.	.	.	T	.	.	.	.	.	A	.	.	.	.	.	.	---	.	.	.	.	.	.	.			.	.	.	.	.	.	.	.	.	.	.	.	.	.	.	.	.
311occult	.	.	.	.	.	.	.	.	M	.	.	.	.	.	.	.	.	.	.	.	.	.	.	.	.	.	.	.	.	.	.			.	.	.	.	.	.	.	N	.	.	.	.	.	.	.	.	.
412occult	.	.	.	.	.	.	.	.	.	.	.	.	.	.	.	.	.	.	.	.	.	.	.	.	.	.	.	.	.	.	.			.	.	.	.	.	.	.	.	.	.	.	.	.	.	.	.	.

Blue = amino acid variations exclusively detected in OBI sequences

Yellow = relevant amino acid substitutions discussed in the manuscript

3b) Pre-C/C region of the 35 HBV/E whole genome sequences:

	Pre-Core				Core																														
Site	5	12	28	29	12	13	21	28	34	38	45	55	64	67	69	74	79	80	83	92	96	101	105	108	109	114	116	130	135	146	147	151	156	180	181	182
Consensus	H	C	W	G	T	V	S	R	A	Y	P	L	E	T	A	V	P	A	D	N	K	L	I	L	T	T	I	P	P	N	T	R	P	A	S	Q
177	.	.	.	.	.	.	.	.	.	.	.	.	.	.	.	.	.	.	.	.	.	.	.	.	.	.	.	.	.	.	.	.	.	.	.	.
188	.	.	.	.	.	.	.	.	.	.	.	.	.	.	.	.	.	.	.	.	.	.	.	.	.	.	.	.	.	.	.	.	.	.	.	.
190	.	.	.	.	.	.	.	.	.	.	.	.	.	.	.	.	.	.	.	.	.	.	.	.	.	.	.	.	.	.	.	.	.	.	.	.
193	.	.	.	.	.	.	.	.	.	.	.	.	.	.	.	.	.	.	.	.	.	.	.	.	.	.	.	.	.	.	.	.	.	.	.	.
199	.	.	.	.	.	.	.	.	.	.	.	.	.	.	.	.	.	.	.	.	.	.	.	.	.	.	.	.	.	.	.	.	.	.	.	.
200	.	.	.	.	.	.	.	.	.	.	.	.	.	.	.	.	.	.	.	.	.	.	.	.	.	.	.	.	.	.	.	.	.	.	.	.
201	.	.	.	.	.	.	.	.	.	.	.	.	.	.	.	.	.	.	.	.	.	.	.	.	.	.	.	.	.	.	.	.	.	.	.	.
209	.	.	.	.	.	.	.	.	.	.	.	.	.	.	.	.	.	.	.	.	.	.	.	.	.	.	.	.	.	.	.	.	.	.	.	.
210	.	.	.	.	.	.	.	.	.	.	.	.	.	.	.	.	.	.	.	.	.	.	.	.	.	.	.	.	.	.	.	.	.	.	.	.
226	.	.	.	.	.	.	.	.	.	.	.	.	.	.	.	.	.	.	.	.	.	.	.	.	.	.	.	.	.	.	.	.	.	.	.	.
232	.	.	.	.	.	.	.	.	.	.	.	.	.	.	.	.	.	.	.	.	.	.	.	.	.	.	.	.	.	.	.	.	.	.	.	.
239	.	.	.	.	.	.	.	.	.	.	.	.	.	.	.	.	.	.	.	.	.	.	.	.	.	.	.	.	.	.	.	.	.	.	.	.
240	D	.	.	D	.	.	.	.	.	.	S	.	D	N	G	G	.	.	.	.	.	.	.	.	.	.	.	Q	.	T	S	.	.	.	.	.
304	.	.	.	.	.	.	.	.	.	.	.	.	.	.	.	.	.	.	.	.	.	.	.	.	.	.	.	.	.	.	.	.	.	.	.	.
306	.	.	.	.	.	.	.	.	.	.	.	.	.	.	.	.	.	.	.	.	.	.	.	.	.	.	.	.	.	.	.	.	.	.	.	.
310	.	.	.	.	.	.	.	.	.	.	.	.	.	.	.	.	.	.	.	.	.	.	.	.	.	.	.	.	.	.	.	.	.	.	.	.
333	.	.	.	.	.	.	.	.	.	.	.	.	.	.	.	.	.	.	.	.	.	.	.	.	.	.	.	.	.	.	.	.	.	.	.	.
334	.	.	.	.	.	.	.	.	.	.	.	.	.	.	.	.	.	.	.	.	.	.	.	.	.	.	.	.	.	.	.	.	.	.	.	.
398	.	.	.	.	.	.	.	.	.	.	.	.	D	.	.	.	.	.	.	.	.	.	.	.	.	.	.	Q	.	T	.	.	.	.	.	.
400	.	R	.	.	.	.	.	.	.	.	.	.	.	.	.	.	.	.	.	.	.	.	.	.	.	.	.	.	.	.	.	.	.	.	.	.
401	.	.	.	.	.	.	.	.	.	.	.	.	.	.	.	.	.	.	.	.	.	.	.	.	.	.	.	.	.	.	.	.	.	.	.	.
402	.	.	.	.	.	.	.	.	.	.	.	.	.	.	.	.	.	.	.	.	.	.	.	.	.	.	.	.	.	.	.	.	.	.	.	.
408	.	.	.	.	.	.	.	.	.	.	.	.	.	.	.	.	.	.	.	.	.	.	.	.	.	.	.	.	.	.	.	.	.	.	.	.
416	.	.	.	.	.	.	.	.	.	.	.	.	.	.	.	.	.	.	.	.	.	.	.	.	.	.	.	.	.	.	.	.	.	.	.	.
438	.	.	.	.	.	.	.	.	.	.	.	.	.	.	.	.	.	.	.	.	.	.	.	.	.	.	.	.	.	.	.	.	.	.	A	.
439	.	.	.	.	.	.	.	.	.	.	.	.	.	.	.	.	.	.	.	.	.	.	.	.	.	.	.	.	.	.	.	.	.	.	A	.
458	.	.	.	.	.	.	.	.	.	.	.	.	.	.	.	.	.	.	.	.	.	.	.	.	.	.	.	.	.	.	.	.	.	.	.	.
472	.	.	*	D	S	.	.	.	.	F	.	.	.	.	.	A	.	G	E	H	.	M	.	.	.	L	V	.	Q	T	A	S	S	.	P	R
476	.	.	.	.	.	.	.	.	.	.	.	.	.	.	.	.	.	.	.	.	.	.	.	.	.	.	.	.	.	.	.	.	.	.	.	.
490	.	.	.	.	.	.	.	.	.	.	.	.	.	.	.	.	.	.	.	.	.	.	.	.	.	.	.	.	.	.	.	.	.	.	.	.
522	.	.	*	D	.	.	P	K	I	F	.	I	N	N	.	.	Q	.	.	.	Q	.	M	I	I	.	L	.	.	T	.	.	.	S	.	.
523	.	.	.	.	.	.	.	.	.	.	.	.	.	.	.	.	.	.	.	.	.	.	.	.	.	.	.	.	.	.	.	.	.	.	.	.
619		.	.	.	.	E	.	.	.	.	.	.	.	.	.	.	.	.	.	.	.	.	.	.	.	.	.	.	.	.	.	.	.	.	.	.
623		.	.	.	.	.	.	.	.	.	.	.	.	.	.	.	.	.	.	.	.	.	.	.	.	.	.	.	.	.	.	.	.	.	.	.
668		.	.	.	.	.	.	.	.	.	.	.	.	.	.	.	.	.	.	.	.	.	.	.	.	.	.	.	.	.	.	.	.	.	.	.

3c) X region of the 35 HBV/E whole genome sequences:

Site	12	22	25	30	34	35	36	43	48	52	73	87	88	94	114	117	123	130	131	146	151
Consensus	A	S	S	V	L	G	D	S	D	H	F	Q	I	H	D	F	L	K	V	A	F
177	.	.	.	.	.	.	.	T	.	.	.	.	L	.	.	.	S	.	.	.	.
188	.	.	.	.	.	.	.	T	.	.	.	.	L	.	.	.	S	.	.	.	.
190	.	.	.	.	.	.	.	T	.	.	.	.	L	.	.	.	S	.	.	.	.
193	.	G	.	I	.	.	.	.	.	.	.	.	L	.	.	.	S	.	.	.	.
199	.	.	.	.	.	.	.	T	.	.	.	.	L	.	.	.	S	.	.	.	.
200	.	.	.	.	.	.	.	T	.	.	.	.	L	.	.	.	S	.	.	.	.
201	.	.	.	.	.	.	.	T	.	.	.	.	L	.	.	.	S	.	.	.	.
209	.	.	.	.	.	.	G	.	.	.	.	.	.	.	.	.	.	.	.	.	.
210	.	.	.	.	.	.	G	.	.	.	.	.	.	.	.	.	.	.	.	S	.
226	.	.	.	.	P	.	.	.	V	.	.	.	.	.	.	.	.	.	.	.	.
232	.	.	.	.	.	.	.	T	.	.	.	.	L	.	.	.	S	.	.	.	.
239	.	.	.	.	.	.	G	.	.	.	.	.	.	.	.	.	.	.	.	.	.
240	.	.	.	.	.	.	.	.	.	.	.	R	L	.	.	.	S	M	I	.	L
304	.	G	.	I	.	.	.	.	.	.	.	.	L	.	.	.	S	.	.	.	.
306	.	.	.	.	P	.	.	.	V	.	.	.	.	.	.	.	.	.	.	.	.
310	.	.	.	.	.	.	.	.	.	N	.	.	L	.	.	.	S	.	.	.	.
333	.	.	P	.	.	.	.	.	.	.	.	.	.	.	.	.	.	.	.	.	.
334	.	.	P	.	.	.	.	.	.	.	.	.	.	.	.	.	.	.	.	.	.
398	.	.	.	.	.	.	G	.	.	.	.	.	.	Y	N	.	.	M	I	.	.
400	.	.	.	.	.	.	G	.	.	.	.	.	.	.	N	.	.	.	.	.	.
401	.	.	.	.	.	.	G	.	.	.	.	.	.	.	N	.	.	.	.	.	.
402	.	.	.	.	.	.	G	.	.	.	.	.	.	.	N	.	.	.	.	.	.
408	.	.	.	.	.	.	G	.	.	.	.	.	.	.	.	.	.	.	.	.	.
416	.	.	.	.	.	.	.	.	.	.	.	.	.	.	.	.	.	.	.	.	.
438	.	.	.	.	.	R	.	.	.	.	.	.	L	.	.	.	S	.	.	.	.
439	.	.	.	.	.	R	.	.	.	.	.	.	L	.	.	.	S	.	.	.	.
458	.	G	.	.	.	.	.	.	.	.	.	.	L	.	.	.	S	.	.	.	.
472	S	.	.	.	.	.	G	.	.	.	V	.	.	.	.	.	.	.	.	.	.
476	.	.	.	.	P	.	.	.	V	.	.	.	.	.	.	.	.	.	.	.	.
490	.	.	.	.	.	.	G	.	.	.	.	.	.	.	.	.	.	.	.	.	.
522	.	.	.	.	.	.	G	.	.	.	.	.	.	.	.	L	S	.	.	.	.
523	.	.	.	.	P	.	.	.	V	.	.	.	.	.	.	.	.	.	.	.	.
619	.	N	.	.	.	.	.	.	.	.	.	.	L	.	.	.	S	.	.	.	.
623	.	.	.	.	.	.	.	T	.	.	.	.	L	.	.	.	S	.	.	.	.
668	.	.	P	.	.	.	.	.	.	.	.	.	.	.	.	.	.	.	.	.	.

3d) P region of the 35 HBV/E whole genome sequences:

Site	9	11	16	39	44	47	67	69	90	108	111	123	169	178	183	188	195	197	213	214	218	220	233	234	247	250	258	265	267	269	272	280	288	295	299	304	305	309	312	316	317	318	319	320	321	336	352	383	436
Consensus	R	I	E	E	Q	N	I	V	N	N	M	L	F	E	A	S	S	H	I	Q	Q	S	R	S	H	T	P	K	V	S	A	V	H	S	H	H	N	S	G	K	R	P	V	F	S	Y	T	A	L
177	.	.	.	.	.	.	.	.	.	.	.	.	C	.	.	.	.	.	.	.	.	.	.	.	.	.	.	.	I	N	.	.	.	.	.	.	S	.	.	.	.	.	.	---	.	.	.	.	.
188	.	.	.	.	.	.	.	.	.	.	.	.	C	.	.	.	.	.	.	.	.	.	.	.	.	.	.	.	I	N	.	.	.	.	.	.	S	.	.	.	.	.	.	---	.	.	.	.	.
190	.	.	.	.	.	.	.	.	.	.	.	.	C	.	.	.	.	.	.	.	.	.	.	.	.	.	.	.	I	N	.	.	.	.	.	.	S	.	.	.	.	.	.	---	.	.	.	.	.
193	.	.	D	.	.	.	.	.	.	.	.	.	C	.	.	.	.	.	.	.	.	.	.	.	.	.	.	.	S	.	.	.	.	.	.	.	.	.	.	.	.	.	.	.	.	.	.	.	.
199	.	.	.	.	.	.	.	.	.	.	.	.	C	.	.	.	.	.	.	.	.	.	.	.	.	.	.	.	I	N	.	.	.	.	.	.	S	.	.	.	.	.	.	---	.	.	.	.	.
200	.	.	.	.	.	.	.	.	.	.	.	.	C	.	.	.	.	.	.	.	.	.	.	.	.	.	.	.	I	N	.	.	.	.	.	.	S	.	.	.	.	.	.	---	.	.	.	.	.
201	.	.	.	.	.	.	.	.	.	.	.	.	C	.	.	.	.	.	.	.	.	.	.	.	.	.	.	.	I	N	.	.	.	.	.	.	S	.	.	.	.	.	.	---	.	.	.	.	.
209	.	.	.	.	.	.	.	.	.	.	.	.	.	.	.	.	.	.	L	.	.	.	.	.	.	.	.	.	.	.	.	.	N	.	.	.	.	.	.	.	.	.	.	.	.	.	.	.	.
210	.	.	.	.	.	.	.	.	.	.	.	.	.	.	.	.	.	.	L	.	.	.	.	.	.	.	.	.	.	.	.	.	.	.	.	.	.	.	.	.	.	.	.	.	.	.	.	.	.
226	.	.	.	.	.	.	T	I	.	.	.	.	.	.	.	.	.	.	.	.	.	.	.	.	.	P	.	.	.	.	.	.	.	.	.	.	.	.	.	.	.	.	.	.	.	.	.	.	.
232	.	.	.	.	.	.	.	.	.	.	.	.	C	.	.	.	.	.	.	.	.	.	.	.	.	.	.	.	I	N	.	.	.	.	.	.	S	.	.	.	.	.	.	---	.	.	.	.	.
239	.	.	.	.	.	.	.	.	.	.	.	.	.	.	.	.	.	.	L	.	.	.	.	.	.	.	.	.	.	.	.	.	N	.	.	.	.	.	.	.	.	.	.	.	.	.	.	.	.
240	.	L	.	.	.	.	.	.	.	.	.	.	C	.	.	.	.	.	.	.	.	.	.	.	.	.	H	N	I	.	.	.	.	P	.	.	.	.	.	.	---	---	---	---	.	.	.	E	.
304	.	.	D	.	.	.	.	.	.	.	.	.	C	.	.	.	.	.	.	.	.	.	.	.	.	.	.	.	S	.	.	.	.	.	.	.	.	.	.	.	.	.	.	.	.	.	.	.	.
306	.	.	.	.	.	.	.	I	.	.	.	.	.	.	.	.	.	.	.	.	.	.	.	.	.	P	.	.	.	.	.	.	.	.	.	.	.	.	.	.	.	.	.	.	.	F	.	.	.
310	.	.	.	.	.	.	.	.	.	.	.	.	C	D	.	.	.	.	.	.	.	.	.	.	.	.	.	.	I	.	.	.	.	.	.	.	.	N	R	.	.	.	.	.	.	.	A	.	.
333	.	.	.	.	.	.	.	.	.	.	.	.	.	.	.	.	L	.	.	.	.	.	.	.	.	.	.	.	.	.	.	.	.	.	.	.	.	.	.	.	.	.	.	.	.	.	.	.	.
334	.	.	.	.	.	.	.	.	.	.	.	.	.	.	.	.	L	.	.	.	.	.	.	.	.	.	.	.	.	.	.	.	.	.	.	.	.	.	.	.	.	.	.	.	.	.	.	.	.
398	.	L	.	.	.	.	L	.	.	.	.	.	.	.	.	.	.	.	.	.	.	.	.	.	.	.	.	.	.	.	.	.	.	.	.	.	.	.	.	---	---	---	---	---	---	.	.	.	.
400	.	.	.	.	.	.	L	.	.	.	.	.	.	.	.	.	.	.	.	.	.	.	.	.	.	.	.	.	.	.	.	.	.	.	.	.	.	.	.	.	.	.	.	.	.	.	.	.	.
401	.	.	.	.	.	.	L	.	.	.	.	.	.	.	.	.	.	.	.	.	.	.	.	.	.	.	.	.	.	.	.	.	.	.	.	.	.	.	.	.	.	.	.	.	.	.	.	.	.
402	.	.	.	.	.	.	L	.	.	.	.	.	.	.	.	.	.	.	.	.	.	.	.	.	.	.	.	.	.	.	.	.	.	.	.	.	.	.	.	.	.	.	.	.	.	.	.	.	.
408	.	.	.	.	.	.	.	.	.	.	.	.	.	.	.	.	.	.	L	.	.	.	.	.	.	.	.	.	.	.	.	.	.	.	.	.	.	.	.	.	.	.	.	.	.	.	.	.	.
416	.	.	.	.	.	.	.	.	H	.	.	.	.	.	.	.	.	.	.	P	P	.	.	.	Y	.	.	.	I	.	.	.	.	.	.	.	.	.	.	.	.	.	.	.	.	.	.	.	I
438	.	.	.	.	.	.	.	.	.	.	.	.	C	.	.	.	.	Y	.	.	.	.	.	.	.	.	.	.	I	.	.	.	.	A	.	.	.	.	.	.	.	.	.	.	.	.	.	.	.
439	.	.	.	.	.	.	.	.	.	.	.	.	C	.	.	.	.	Y	.	.	.	.	.	.	.	.	.	.	I	.	.	.	.	A	.	.	.	.	.	.	.	.	.	.	.	.	.	.	.
458	.	.	D	.	.	.	.	.	.	.	.	.	C	.	.	.	.	.	.	.	.	.	.	.	.	.	.	.	S	.	.	.	.	.	.	.	.	.	.	.	.	.	.	.	.	.	.	.	.
472	.	L	Q	.	.	D	.	.	.	.	.	.	.	.	.	.	.	.	.	.	.	P	.	.	.	.	.	.	.	.	.	.	.	.	.	.	.	.	.	.	.	.	.	.	.	.	.	.	I
476	.	.	.	.	.	.	.	I	.	.	.	.	.	.	.	.	.	.	.	.	.	.	.	.	.	P	.	.	.	.	.	.	.	.	.	.	.	.	.	.	.	.	.	.	.	.	.	.	.
490	.	.	.	.	.	.	.	.	.	.	.	.	.	.	T	.	.	.	.	.	.	.	.	.	.	.	.	.	.	.	.	.	.	.	.	.	.	.	.	.	.	.	.	.	.	.	.	.	.
522	L	L	.	D	H	.	.	.	.	K	L	.	.	.	T	S	.	.	.	.	.	.	T	G	.	.	.	.	.	.	S	.	.	.	R	.	.	.	.	.	.	.	.	.	.	.	.	.	.
523	.	.	.	.	.	.	.	I	.	.	.	.	.	.	.	.	.	.	.	.	.	.	.	.	.	P	.	.	.	.	.	.	.	.	.	.	.	.	.	.	.	.	.	.	.	.	.	.	.
619	.	.	.	.	.	.	.	.	.	.	.	F	C	.	.	.	.	.	.	.	.	.	.	.	.	.	.	.	I	.	.	I	.	.	.	P	.	.	.	.	.	.	.	.	.	.	.	.	.
623	.	.	.	.	.	.	.	.	.	.	.	.	C	.	.	.	.	.	.	.	.	.	.	.	.	.	.	.	I	N	.	.	.	.	.	.	S	.	.	.	.	.	.	.	.	.	.	.	.
668	.	.	.	.	.	.	.	.	.	.	.	.	.	.	.	.	L	.	.	.	.	.	.	.	.	.	.	.	.	.	.	.	.	.	.	.	.	.	.	.	.	.	.	---	.	.	.	.	.

Site	462	465	479	480	568	579	582	593	602	611	612	635	658	660	662	678	681	682	691	709	714	742	782	796	797	806	808	824	827
Consensus	S	R	D	S	S	H	P	N	W	R	M	I	A	I	S	K	L	N	P	I	M	K	A	I	Y	Q	T	H	D
177	.	.	.	.	.	.	.	H	.	.	L	.	.	T	.	.	.	.	.	.	.	.	.	.	C	.	.	.	.
188	.	.	.	.	.	.	.	H	.	.	L	.	.	T	.	.	.	.	.	.	.	.	.	.	C	.	.	.	.
190	.	.	.	.	.	.	.	H	.	.	L	.	.	T	.	.	.	.	.	.	.	.	.	.	C	.	.	.	.
193	.	.	.	.	.	.	.	.	.	.	L	.	.	T	.	.	.	.	.	.	.	.	.	.	.	.	.	.	V
199	.	.	.	.	.	.	.	H	.	.	L	.	.	T	.	.	.	.	.	.	.	.	.	.	C	.	.	.	.
200	.	.	.	.	.	.	.	H	.	.	L	.	.	T	.	.	.	.	.	.	.	.	.	.	C	.	.	.	.
201	.	.	.	.	.	.	.	H	.	.	L	.	.	T	.	.	.	.	.	.	.	.	.	.	C	.	.	.	.
209	.	.	.	.	.	.	.	.	.	K	.	L	.	.	.	.	.	.	Q	.	.	.	.	V	.	R	.	.	.
210	.	.	.	.	.	.	.	.	.	K	.	L	.	.	.	.	.	.	Q	.	.	.	.	V	.	R	.	.	.
226	.	.	.	.	.	.	.	H	.	K	.	.	.	.	A	.	.	.	.	.	.	.	.	.	.	.	S	.	.
232	.	.	.	.	.	.	.	H	.	.	L	.	.	T	.	.	.	.	.	.	.	.	.	.	C	.	.	.	.
239	.	.	.	.	.	.	.	.	.	K	.	L	.	.	.	.	.	.	Q	.	.	.	.	V	.	R	.	.	.
240	.	.	.	.	.	.	.	.	F	.	L	.	.	.	.	.	.	.	.	.	.	.	.	.	.	.	.	.	.
304	.	.	.	.	.	.	.	.	.	.	L	.	.	T	.	.	.	.	.	.	.	.	.	.	.	.	.	.	V
306	.	.	.	.	.	.	.	H	.	K	.	.	.	.	A	.	.	.	.	.	.	.	.	.	.	.	S	.	.
310	.	.	.	.	A	.	.	.	.	.	L	.	T	.	.	.	.	.	S	.	.	.	.	.	.	.	.	.	.
333	.	.	.	.	.	.	S	H	.	.	.	.	.	.	.	Q	.	.	.	.	.	.	.	.	.	.	.	R	V
334	.	.	.	.	.	.	S	H	.	.	.	.	.	.	.	Q	.	.	.	.	.	.	.	.	.	.	.	R	V
398	.	.	.	Y	.	.	.	.	.	K	.	.	T	.	Y	Q	.	.	.	.	.	T	.	V	.	.	.	.	.
400	.	.	.	.	.	.	.	.	.	K	.	.	T	.	Y	.	.	.	.	.	V	T	.	V	.	.	.	.	.
401	.	.	.	.	.	.	.	.	.	K	.	.	T	.	Y	.	.	.	.	.	V	T	.	V	.	.	.	.	.
402	.	.	.	.	.	.	.	.	.	K	.	.	T	.	Y	.	.	.	.	.	V	T	.	V	.	.	.	.	.
408	.	.	.	.	.	.	.	.	.	K	.	L	.	.	.	.	.	.	Q	.	.	.	.	V	.	R	.	.	.
416	.	.	N	.	.	Q	A	.	.	.	.	.	.	.	.	.	Q	Y	.	.	.	.	.	.	.	.	.	.	.
438	.	.	.	.	.	.	.	.	.	K	L	.	.	.	.	.	.	.	.	.	.	.	.	.	.	.	.	.	.
439	.	.	.	.	.	.	.	.	.	K	L	.	.	.	.	.	.	.	.	.	.	.	.	.	.	.	.	.	.
458	.	.	.	.	.	.	.	.	.	.	L	.	.	.	.	.	.	.	.	.	.	.	.	.	.	.	.	.	V
472	P	K	.	.	.	.	.	.	.	K	.	.	.	.	.	.	.	.	.	.	.	T	.	V	C	.	.	.	V
476	.	.	.	.	.	.	.	H	.	K	.	.	.	.	A	.	.	.	.	.	.	.	.	.	.	.	S	.	.
490	.	.	.	.	.	.	.	.	.	K	.	.	.	.	.	.	.	.	.	.	.	.	.	V	.	.	.	.	.
522	.	.	.	.	.	.	.	.	.	K	.	.	.	.	.	Q	R	.	.	.	.	Q	.	V	.	.	.	.	.
523	.	.	.	.	.	.	.	H	.	K	.	.	.	.	A	.	.	.	.	.	.	.	.	.	.	.	S	.	.
619	.	.	.	.	A	.	.	.	.	.	L	.	.	T	.	.	M	.	.	M	.	.	T	.	.	.	.	.	.
623	.	.	.	.	.	.	.	H	.	.	L	.	.	T	.	.	.	.	.	.	.	.	.	.	C	.	.	.	.
668	.	.	.	.	.	.	S	H	.	.	.	.	.	T	.	Q	.	.	.	.	.	.	.	.	.	.	.	.R	V

3e) Pre-S/S region of the 7 HBV/A sequences as well as the 2 OBI HBV/A pre-S/S sequences:

	Pre-S1						Pre-S2									S-region							
Site	5	73	78	97	102	114	1	7	19	22	24	28	35	37	38	4	45	106	194	200	207	217	224
Consensus	L	G	S	T	G	E	M	T	G	F	A	N	V	T	T	I	S	V	V	Y	S	P	A
203	I	.	T	A	E	D	I	P	.	.	.	S	A	N	I	T	T	.	A	.	N	.	V
260	.	.	.	.	.	D	.	.	.	.	V	.	.	N	.	.	.	.	.	F	N	.	.
299	.	.	.	.	.	.	.	.	.	.	.	.	.	.	.	.	.	.	.	.	.	.	.
420	.	.	.	.	.	.	.	.	.	.	.	.	.	.	.	.	.	.	.	.	.	.	.
443	.	.	.	.	.	.	.	.	.	.	.	.	.	.	.	.	.	.	.	.	.	.	.
446	.	.	.	.	.	.	.	.	.	.	.	.	.	.	.	.	.	.	.	.	.	.	.
471	.	S	.	.	.	D	.	.	S	L	V	.	.	N	.	.	.	.	.	.	R	L	V
296occult	.	.	.	.	.	.	.	.	.	.	.	.	.	.	.	.	.	I	.	.	.	.	.
405occult	.	.	.	.	.	.	.	.	.	.	.	.	.	.	.	.	.	I	.	.	.	.	.

Blue = amino acid variation exclusively detected in OBI sequences

Yellow = relevant amino acid substitutions discussed in the manuscript
